# The application of mirabilite in traditional Chinese medicine and its chemical constituents, processing methods, pharmacology, toxicology and clinical research

**DOI:** 10.3389/fphar.2023.1293097

**Published:** 2024-01-04

**Authors:** Lianbo Tao, Jiaqing Fu, Fangjie Wang, Yinglian Song, Yi Li, Jingwen Zhang, Zhang Wang

**Affiliations:** ^1^ College of Ethnomedicine, Chengdu University of Traditional Chinese Medicine, Chengdu, China; ^2^ State Key Laboratory of Southwestern Chinese Medicine Resources, Chengdu University of Traditional Chinese Medicine, Chengdu, China; ^3^ College of Pharmacy, Chengdu University of Traditional Chinese Medicine, Chengdu, China

**Keywords:** mirabilite, traditional medicine, processing methods, pharmacology, toxicology, clinical applications

## Abstract

**Purpose:** This study reviews the use of mirabilite in traditional Chinese medicine and various preparations by describing its chemical composition, processing methods, pharmacology, toxicology, and clinical research progress.

**Methods:** The applications and processing methods of mirabilite are searched in traditional and modern Chinese medical writings, and the articles on chemical composition, pharmacological effects, toxicology, and clinical studies of mirabilite and its combinations in PubMed and China Knowledge Network are reviewed, sorted, and analyzed.

**Results:** The main chemical component of mirabilite is sodium sulfate decahydrate (Na_2_SO_4_·10H_2_O), followed by small amounts of sodium chloride, magnesium sulfate, calcium sulfate, and other inorganic salts. This study systematically organizes the history of the medicinal use of mirabilite in China for more than 2,000 years. This mineral has been used by nine Chinese ethnic groups (Han, Dai, Kazakh, Manchu, Mongolian, Tujia, Wei, Yi, and Tibetan) in a large number of prescription preparations. The Pharmacopoeia of the People’s Republic of China (2020 edition) records stated that mirabilite can be used for abdominal distension, abdominal pain, constipation, intestinal carbuncle, external treatment of breast carbuncle, hemorrhoids, and other diseases. The traditional processing methods of mirabilite in China include refining, boiling, sautéing, filtration after hot water blistering, and firing. Since the Ming Dynasty, processing by radish has become the mainstream prepared method of mirabilite. Mirabilite can exhibit anti-inflammatory detumescence effects by inhibiting AMS, LPS, IL-6, IL-10, TNF-α, and NO levels and attenuating the upregulation of TNF-α and NF-κB genes. It can promote cell proliferation and wound healing by increasing the production of cytokines TGFβ1 and VEGF-A and gastrointestinal motility by increasing the release of vasoactive intestinal peptide, substance P, and motilin. It can increase the expression of low-density lipoprotein receptor and AKT phosphorylation in the liver by up-regulating bile acid synthesis genes; reduce TRB3 expression in the liver, FGF15 co-receptor KLB expression, and FGF15 production in the ileum, and JNK signal transduction; and increase the transcription of CYP7A1 to achieve a cholesterol-lowering effect. Mirabilite also has a variety of pharmacological effects, such as regulating intestinal flora, anti-muscle paralysis, anti-colon cancer, promoting water discharge, and analgesic. Only a few toxicological studies on mirabilite are available. External application of mirabilite can cause local skin to be flushed or itchy, and its oral administration is toxic to neuromuscular cells. The sulfur ions of its metabolites can also be toxic to the human body. At present, no pharmacokinetic study has been conducted on mirabilite as a single drug. This mineral has been widely used in the clinical treatment of inflammation, edema, wound healing, digestive system diseases, infusion extravasation, hemorrhoids, skin diseases, breast accumulation, muscle paralysis, intestinal preparation before microscopic examination, and other diseases and symptoms.

**Conclusion:** Mirabilite has good application prospects in traditional Chinese medicine and ethnomedicine. In-depth research on its processing methods, active ingredients, quality control, pharmacokinetics, pharmacological and toxicological mechanisms, and standardized clinical application is needed. This paper provides a reference for the application and research of mirabilite in the future.

## Graphical Abstract

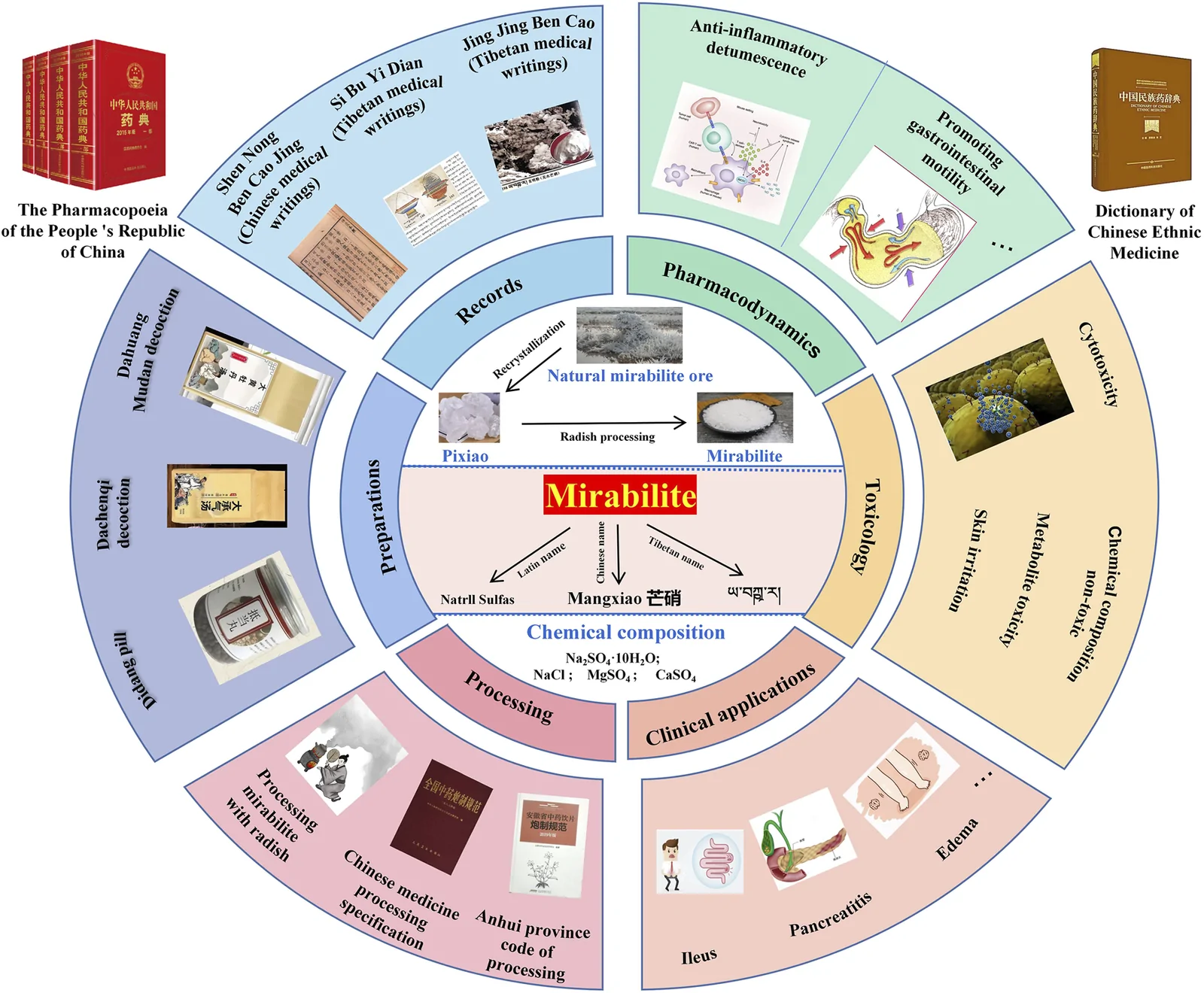

## 1 Introduction

Mirabilite is a salt sediment that mainly appears in the form of aqueous mirabilite and anhydrous mirabilite and combines with other compounds to form complex salts, such as calcium mirabilite, potassium mirabilite, and carbonate mirabilite (Li, 2020). More than 10 kinds of mirabilite exist in nature; among which, sodium mirabilite (i.e., ten water mirabilite and anhydrous mirabilite) and calcium mirabilite (Shen, 2003) have industrial utilization values. Ten water mirabilite is formed through the deposition of sulfate minerals in a dry and cold environment; the colder the climate, the easier the deposition and the thicker the deposit. Anhydrous mirabilite is deposited at temperatures >10° warmer than those for natural mirabilite (Li, 2020). By conducting Aral Sea brine evaporation tests and Ferghana salt system studies, some researchers proposed that calcium mirabilite is crystallized directly from sulfate (Wei, 2001).

Mirabilite is a basic chemical substance commonly found in nature, and its formation is closely related to that of saline lake water, paleoclimate, paleoenvironment, and their related metallic and nonmetallic deposits (Ren, 2013). China has extremely rich mirabilite resources, and its total proven reserves of mirabilite are more than 20 billion tons (calculated by Na_2_SO_4_), ranking first in the world. At the end of 2018, 167 mines with proven reserves and 256 mineral production sites were found across 18 provinces (regions) in China (Li, 2020). These reserves are relatively concentrated in various provinces, with greater than 1 billion tons found in Qinghai, Sichuan, Hunan, and Inner Mongolia, accounting for 90% of the national production (Ren, 2013). Sichuan has the richest resources of calcium mirabilite with high quality (the mass fraction of sodium sulfate in the ore is 30%–50%). Owing to its stable and reliable resources, simple geological structure, and good recoverability, it has gradually become the largest mirabilite producer and exporter in China. According to the type of mineralization, China’s mirabilite ores are divided into modern inland salt-lake mirabilite deposits and ancient inland salt-lake calcium mirabilite deposits. The former are mostly distributed in the north of the Qinling Mountains, Xinjiang, Qinghai, Ningxia, Gansu, Inner Mongolia, Heilongjiang, Tibet, and other vast plateau or desert arid climate zones in the salt lake. Meanwhile, the majority of ancient inland salt-lake calcium mirabilite deposits are distributed in Sichuan, Yunnan, Hubei, Hunan, Anhui, Xinjiang, Gansu, and other provinces (regions). Sodium mirabilite is mostly distributed in modern salt-lake deposits, and calcium mirabilite is mostly obtained from ancient salt-lake deposits (Li, 2020).

Mirabilite (Latin name: *Natrll Sulfas*; Tibetan name: 
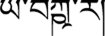
) is a mineral in traditional Chinese medicine that shows a prismatic structure and appears as oblong or irregular lumps and grains that are colorless, transparent or off-white translucent, brittle, and friable, with glassy luster on the cross-section (Wang, 2014). Its main ingredient is aqueous sodium sulfate, which is salty, bitter, and characteristically cold and is mainly used for accumulation, and constipation, with the efficacy of clearing away heat and fire, resolving blood stasis, and dispersing knots (Huang and Zhang, 2008). Mirabilite has been used medicinally in China for more than 2,000 years. As early as the Han Dynasty (202–220 BC), the name Poxiao (mirabilite) was documented and recorded in the upper class of medicines during the period of the Shen Nong Ben Cao Jing (Shang, 1981), and the efficacy of mirabilite was summarized in many subsequent books on ethnomedicine. Many preparations of mirabilite have been developed, the most common of which is Dachenqi decoction that has been studied in depth. Dachenqi decoction can inhibit the production of IL-1, IL-6, and TNF-α to achieve anti-inflammatory effects, rapidly reduce blood lipids by blocking the peroxisome proliferator-activated receptor γ pathway (Liu et al., 2020), and reduce blood lipids by lowering the levels of serum diamine oxidase (DAO), tumor necrosis factor-α (TNF-α), and interleukin-8 (IL-8) and elevating the gastric motility factor (MTL) for the treatment of intestinal obstruction (Gai et al., 2023). In addition, Dachenqi decoction has laxative (Zhao et al., 2013), antibacterial (Hu et al., 1999), anti-endotoxin (Tian et al., 1998; Sun et al., 2011), antipyretic (Zhang and Yang, 2009), detoxification (Liu, 2010) effects. It also has a significant effect on gastrointestinal (Zhang et al., 2011), immune (Yang et al., 2004; Guo and Bi, 2011), and digestive (Zhang, et al., 1998) functions and has a significant protective effect on the brain, lungs, and other important organs (Li et al., 2002). Owing to the similarity of efficacy between *Rheum palmatum* and mirabilite, their combination is the most commonly for treatments, such as for abdominal distension (Zhang, 2023), female pelvic inflammatory masses (Meng and Tian, 2023), constipation (Wan and Xiao, 2022), wound healing (Li, 2021), phlebitis (Sun Bobo and Pan, 2020), pneumonia (Xiao et al., 2019), and other diseases.

The modern biology of mirabilite production is relatively simple, that is, the natural mirabilite is dissolved in hot water, filtered, and cooled to precipitate crystals, which are commonly known as “Pixiao.” The radish is washed, sliced, placed in a pot with water and Pixiao, and boiled. The upper layer of liquid is discarded, and the precipitation crystals are cooled down and named as mirabilite. Mirabilite prepared by weathering involves losing the water of crystallization into a white powder called Xuanming powder (Chen, 2012). In the modern industry, mirabilite is often produced by brine freezing and separation (Li, 1991). He and Wang (2020) prepared mirabilite particles by antisolvent recrystallization and concluded that the optimum one-factor conditions for the preparation of mirabilite particles were the solvent–antisolvent ratio of 1:4, stirring for 40 min, and droplet acceleration of 2 mL/min. Under these optimum conditions, mirabilite particles were obtained with about 5 m average size, complete hexahedral shapes, and uniform surfaces. Mirabilite has a wide range of uses and has traditionally been utilized in four main industries: paper, detergents, glass, and printing and dyeing. In the 18th century, mirabilite was used as a raw material for the industrial production of soda ash (sodium carbonate) (Li, 1991b). At present, its main use is for the synthesis chemical products such as Yuanming powder (anhydrous sodium sulfate with a purity of more than 99%), alkali sulfide, and sodium silicate. With the continuous progress of science and technology, mirabilite has widely been used in more than 20 industries, such as the chemical industry, light industry, textile, building materials, medicine, nonferrous metallurgy, and leather industry (Zhu, 2014).

With the continuous progress of science and technology, mirabilite has widely been used in more than 20 industries, such as the chemical industry, light industry, textile, building materials, medicine, nonferrous metallurgy, and leather industry (Zhu, 2014).

With the advancement of medical technology and the expansion of the scope of people’s knowledge of traditional Chinese medicine, mirabilite has played an increasingly important role in medical treatment. Therefore, this study will further promote the global use of mirabilite by describing its application in traditional Chinese medicine and its processing methods, chemical composition, pharmacology, toxicology, and clinical studies for future research.

## 2 Chemical composition of mirabilite

The main constituent of mirabilite is sodium sulfate decahydrate (Na_2_SO_4_·10H_2_O), followed by small amounts of inorganic salts such as sodium chloride, magnesium sulfate, and calcium sulfate (Wu et al., 2007). Japan preserved several Tang Dynasty medicines, one of which is “mirabilite,” magnesium sulfate heptahydrate (MgSO_4_·7H_2_O) with the highest purity (Liu, 2001). According to the Japanese Han Fang Ye Wu Zhi Zhen (1981 edition), crystallized magnesium sulfate and aqueous sodium sulfate can be used as mirabilite (Xue, 1991). The Jing Zhu Ben Cao recorded mirabilite as Na_2_SO_4_·10H_2_O containing 19.3% Na_2_O, 3.2%–4.8% SO, and 55.9% H_2_O (Pontso, 2012). Ao (2023) measured the chemical fractions of natural mirabilite in the mirabilite ore of Zemubilite salt in Xinjiang as follows: Na_2_SO_4_, with a content of 16.34%–93.13% and an average of 83.5%; NaCl, with the content of 0.15%–3.5% and an average of 1.05%; and CaSO_4_, with the content of 0.64%–3.12% and an average of 1.6%. Xue (1991) conducted qualitative and quantitative tests on mirabilite purchased from markets all over China by X-ray diffraction, fluorescence X-ray analysis, and heating analysis. They found that the main component of mirabilite was Na_2_SO_4_, accounting for 88%–96%. It also contained traces of elements such as K, Ca, Fe, Cu, Zn, Br, and Sr. The content of Mg was very low at only 300–700 ppm. Song et al. (2020) identified mirabilite, Poxiao (mirabilite), and saltpeter and found that mirabilite mainly contained Na, Mg, K, Fe, and other elements. The average values of Li, Be, Cr, Co, Ni, Ga, Se, Rb, Cs, Ti, and other elements were low, and Ti and Ga had the lowest levels. The average values of Al, Zn, Mn, Sr, and Ba were relatively high.

## 3 Applications of mirabilite in traditional Chinese medicine

### 3.1 Records of mirabilite in traditional and modern Chinese medical writings

Mirabilite has a long history of use in China and is recorded in numerous medical writings (Table 1). It is also known as Pixiao, Poxiao, Penxiao, Yaxiao, and Mayaxiao. Shen Nong Ben Cao Jing first recorded mirabilite with the name of Poxiao and described its characteristic, taste, toxicity, and main treatments (Shang, 1981). Ben Cao Jing Ji Zhu (AD 420–589) recorded the source and collection time of Poxiao (mirabilite). There’s another record of mirabilite’s treatment (Shang, 1994). Zhen Quan’s Yao Xing Lun (AD 608) emphasized the constipation-curing effect of mirabilite and proposed for the first time that mirabilite solution can be used externally to treat skin diseases (Zhen, 1983). Su Jing’s Xin Xiu Ben Cao (AD 657–659) and Tang Shenwei’s Zheng Lei Ben Cao (AD 1082–1098) both inherited the record of Ben Cao Jing Ji Zhu. Zhang Yuansu’s Jie Gu Zhen Zhu Nang (AD 1234) recorded the three major effects of mirabilite: “clear heat, treat constipation, and abdominal mass” (Li, 1991). Li Shizhen’s Ben Cao Gang Mu (AD 1552–1578) summarized the characteristics, taste, main treatment, toxicity, source, and collection time of mirabilite in Shen Nong Ben Cao Jing, Ben Cao Jing Ji Zhu, and Yao Xing Lun (Li, 2014b). Jia Suoxue’s Yao Pin Hua Yi (AD 1644) recorded that mirabilite has a significant effect on treating abdominal mass and clearing heat (Jia, 2015). According to Wang Ang’s Ben Cao Bei Yao (AD 1694), “mirabilite can expel phlegm, unblock collaterals, induce abortions, and resolve stones. It can treat abdominal mass, typhoid fever, epidemic dysentery, accumulation, blood stasis, jaundice, gonorrhea, scrofula, and cataract” (Wang and Zheng, 2019). Tibetan medicine writing Jing Zhu Ben Cao (AD 1736–1796) recorded that mirabilite is a white nitrate salt that is produced in the deep valley of the cave and tastes sweet, similar to wheat flour. It has the functions of raising stomach Yang, helping digestion, and removing tumors. These findings emphasized the main effect of mirabilite on gastrointestinal and other digestive tract diseases (Pontso, 2012). In the modern times, the records of mirabilite have gradually been completed (Yiyu, 1956; Commission, 2019).

**TABLE 1:** Records of traditional and modern Chinese medical writings on mirabilite.

Traditional medicinal writings of China	Years	Records of mirabilite	References
Shen Nong Ben Cao Jing	BC 202-AD 220	Character cold, taste bitter, no poisonous; Treat abdominal mass, stomach distension, dispelling blood stasis, removing dampness and resolving stones	Shang (1981)
Ben Cao Jing Ji Zhu	AD 420-589	Produced on the sunny side of the salt-lakes in Yizhou, collected in all seasons.	Shang (1994)
		Treat abdominal mass, gonorrhea, and stomach distension; clear heat, dispel blood stasis, and remove dampness, unblock collaterals, urination, defecation, menstruation, produced from Poxiao	
Yao Xing Lun	AD 608	Treat women‘s amenorrhea, abdominal mass, scrofula, jaundice, and induce abortion, external application of mirabilite solution can treat skin disease	Zhen (1983)
Jie Gu Zhen Zhu Nang	AD 1234	Clear heat, and treat constipation, and abdominal mass	Li, G. (1991)
Ben Cao Gang Mu	AD 1552–1578	Summarize the contents of the Shen Nong Ben Cao Jing, Ben Cao Jing Ji Zhu and Yao Xing Lun	Li, S. (2014)
Yao Pin Hua Yi	AD 1644	Clear heat, abdominal distension and constipation	Jia (2015)
Ben Cao Bei Yao	AD 1694	Treat abdominal mass, typhoid fever, epidemic dysentery, accumulation, blood stasis, jaundice, gonorrhea, scrofula, pyocutaneous disease, and cataract	Wang and Zheng (2019)
Jing Zhu Ben Cao	AD 1736–1796	Treat stomach cold, indigestion, heart disease, tumor and edema	Pontso (2012)
Chinese Pharmacology Outline	AD 1956	Dispell blood stasis, dampness, unlock collaterals, and induce urination, defecation, menstruation, treat accumulation, abdominal distension, gonorrhea, scrofula, jaundice, fever, abortion, lacquer sores	Yiyu (1956)
The Pharmacopoeia of the People’s Republic of China	AD 2020	Character cold, taste bitter, salty, treat accumulation, abdominal distension, constipation, intestinal carbuncle, and external treatment for breast carbuncle, hemorrhoids	Commission (2019)

### 3.2 Application of mirabilite as a traditional Chinese medicine

Mirabilite is used in traditional Chinese medicine and in eight ethnic groups (Dai, Kazakh, Manchu, Mongolian, Tujia, Wei, Yi, and Tibetan) in China (Table 2). In traditional Chinese medicine and ethnomedicine, mirabilite is mainly used for the treatment of digestive diseases, urological diseases, dermatologic diseases, intestinal carbuncle, breast carbuncle, hemorrhoids, ulcers, abdominal distension, amenorrhea, mouth sores, and toothache with good results. Traditional Chinese medicine and eight ethnic groups of medicine all emphasize the therapeutic effect of mirabilite on constipation. In traditional Chinese and Wei medicine, it is used for the treatment of digestive system diseases and external treatment for breast carbuncle and hemorrhoids. In Mongolian and Tibetan medicine, it is used for the treatment of amenorrhea, indigestion, and edema. In Dai medicine, it is used for the treatment of skin and dental diseases. In Tujia and Yi medicine, it is used for the treatment of sores and eye redness. In Tibetan medicine, it has special application for heart disease and tumor. In Kazakh medicine, it has special application for bad breath. In Manchu medicine, it has special application for epistaxis.

**TABLE 2:** Application of mirabilite in Chinese traditional medicine.

Chinese traditional medicine	Application
Traditional Chinese medicine	Accumulation, abdominal distension, constipation, intestinal carbuncle, and external treatment for breast carbuncle, hemorrhoids
Dai medicine	Skin furuncle, ringworm, itching, patchy rash, scabies, eczema, wormy teeth, swollen and painful gums
Kazakh medicine	Constipation, bad breath
Manchu medicine	Constipation, epistaxis
Mongolian medicine	Many parts of the sense of expansion, amenorrhea, indigestion, edema, bladder stones, closed urine, frequent urination, constipation, breast nodules, erysipelas
Tujia medicine	Abdominal distension, constipation, redness of the eyes, mouth sores, pharyngitis, hemorrhoids
Wei medicine	Constipation, abdominal distension, abdominal pain, intestinal carbuncle, breast carbuncle, hemorrhoids
Yi medicine	Internal and external sores, abdominal distension, stomach pain, gangrene, eye redness and eye pain
Tibetan medicine	Stomach cold, indigestion, constipation, edema, heart disease, tumor, abdominal distension, erysipelas, jaundice, amenorrhea, redness of the eyes and eye pain

Note: The above information is from the Pharmacopoeia of the People’s Republic of China (Commission, 2019) and the Dictionary of Chinese Ethnic medicines (Jia and Zhang, 2016).

### 3.3 Application and statistical analysis of mirabilite in Chinese traditional medicine preparations

According to statistics, mirabilite was used in 44 preparations, including 27 decoctions, 6 pulvis, 5 pills, 2 Dans, and 1 for each of the other dosage forms (e.g., external lotions, buccal tablets, mixtures, and granules). Table 3 shows the application of mirabilite in traditional Chinese medicine preparations, including the dosage form, preparation name, composition and dose of medicinal materials, method use, role of mirabilite in each preparation, and indications.21

**TABLE 3:** Application of mirabilite in traditional Chinese medicine preparations.

Dosage form	Preparation name	Composition and dose of medicinal materials	Using method	The role of mirabilite in each preparation	Indications
Decoctions	Dahuang Mudan decoctions	*Rheum palmatum* (12 g)*, Paeonia suffruticosa* (3 g)*, Persicae semen* (9 g)*, Benincasa hispida* (30 g), mirabilite (9 g)	Internal use	Purgative	Intestinal carbuncle, abdominal pain, fever, spontaneous sweating aversion to cold, acute simple appendicitis, intestinal obstruction, acute biliary tract infection, pancreatitis, acute pelvic inflammatory disease, infection after tubal ligation
	Dachengqi decoctions	*Rheum palmatum* (12 g)*, Houpoea officinalis* (15 g)*, Citrus aurantium* (12 g), mirabilite (9 g)	Internal use	Purgative	Constipation, bloating, abdominal pain, hot flashes, delirium, dry mouth and tongue, hot convulsions, spasms, madness
	Xinjia Huanglong decoctions	*Rehmannia glutinosa* (15 g)*, Glycyrrhiza uralensis* (6 g)*, Panax ginseng* (4.5 g)*, raw Rheum palmatum* (9 g)*,* mirabilite (3 g)*, Scrophularia ningpoensis* (15 g)*, Ophiopogon japonicus* (15 g)*, Angelica sinensis* (4.5 g)*, Stichopus japonicus* (2strips)*, Zingiber officinale* (6spoons)	Internal use	Purgative	Heat syndrome, constipation, abdominal distension, tired, dry mouth and throat, cleft lip and tongue coke
	Tiaowei Chengqi decoctions	*Rheum palmatum* (200 g)*, Glycyrrhiza uralensis* (100 g)*,* mirabilite (85 g)	Internal use	Purgative	Constipation, thirst, upset, fever, abdominal distension, delirium, bleeding, swelling and pain of mouth, teeth and throat
	Daxianxiong decoctions	*Rheum palmatum* (10 g)*,* mirabilite (10 g)*, Euphorbia kansui* (1 g)	Internal use	Purgative	Infracardiac pain, irritability, constipation, dry mouth and dry tongue, hot flashes, acute pancreatitis, acute intestinal obstruction, liver abscess, exudative pleurisy, cholecystitis, cholelithiasis
	Fufang Dachengqi decoctions	*Houpoea officinalis* (15–20 g)*, Raphanus sativus* (15–30 g)*, Citrus aurantium* (15 g)*, Prunus persica* (9 g)*, Paeonia lactiflora* (15 g)*, Rheum palmatum* (9–15 g)*,* mirabilite (9–15 g)	Internal use	Purgative	Simple intestinal obstruction
	Huanglong decoctions	*Rheum palmatum* (9 g)*,* mirabilite (12 g)*, Citrus aurantium* (6 g)*, Houpoea officinalis* (3 g)*, Angelica sinensis* (9 g)*, Panax ginseng* (6 g)*, Glycyrrhiza uralensis* (3 g)	Internal use	Purgative	Chest accumulation, Qi and blood deficiency, constipation, abdominal distension, abdominal pain, body heat and thirst, tired, delirium, fainting limbs, yellow or black tongue coating, typhoid fever, paratyphoid fever, epidemic cerebrospinal meningitis, encephalitis B, senile intestinal obstruction
	Daxianxiong decoctions	*Rheum palmatum* (35 g)*,* mirabilite (15 g)*, Euphorbia kansui* (5 g)	Internal use	Purgative	Typhoid sun syndrome is not resolved, chest heat, heart pain
	Fangzhanlian decoctions	*Lindera aggregata* (12 g)*, MeLia toosendan* (12 g)*, Houpoea officinalis* (9 g)*, Orydalisyanhusuo* (9 g)*, Angelica sinensis* (12 g)*, Paeonia lactiflora* (9 g)*, Citrus aurantium* (9 g)*, Raphanus sativus* (12 g)*, Rheum palmatum* (15 g)*,* mirabilite (6 g)	Internal use	Purgative	Chest accumulation, qi stagnation, blood stasis
	Dacheng decoctions	*Angelica sinensis* (100 g)*, Rheum palmatum* (200 g)*,* mirabilite (100 g), *Akebia quinata* (100 g)*, Biancaea sappan* (100 g)*, Citrus aurantium,* (200 g) *Houpoea officinalis* (a little)*, Carthamus tinctorius* (100 g)*, Citrus reticulata* (100 g)*, Glycyrrhiza uralensis* (100 g)	Internal use	Purgative	Blood stasis is not scattered, bloating, constipation, gonorrhea
	Zengye Chengqi decoctions	*Scrophularia ningpoensis* (50 g)*, Ophiopogon japonicus* (40 g)*, Rehmannia glutinosa* (40 g)*, Rheum palmatum* (15 g)*,* mirabilite (7.5 g)	Internal use	Purgative	Blood deficiency and intestinal dryness constipation syndrome, fluid deficiency
	Wujiapi decoctions	*Angelica sinensis* (10 g)*, Commiphora myrrha* (10 g)*, Acanthopanar gracilistulus* (10 g)*,* mirabilite (10 g), C*itrus reticulata* (10 g)*, Zanthoxylum bungeanum* (10 g)*, Cyperus rotundus* (10 g)*, Syringa oblata* (3 g)*,* moschus (0.3 g), A*llium fistulosum* (3roots)*, Lycium chinense* (3 g)*, Paeonia suffruticosa* (6 g)	External use	Removing dampness	Falling injury, eye swelling
	Sanhuang Paishi decoctions	*Scutellaria baicalensis* (15 g)*, Rheum palmatum* (15 g)*, Gardenia jasminoides* (15 g)*, Artemisia capillaris* (30 g)*, Lysimachia christiniae* (50 g)*, Lonicera japonica* (15 g)*, Curcuma aromatica* (15 g)*, Radix aucklandiae* (10 g)*, Houpoea officinalis* (12 g)*,* mirabilite (10 g)	Internal use	Clearing heat, removing dampness, purgative	Liver and gallbladder damp heat accumulation, gastrointestinal real heat
	Wuwei Xiaodu and Dahuang Mudan decoctions	*Lonicera japonica* (15 g)*, Taraxacum mongolicum* (15 g)*, Chrysanthemum morifolium* (15 g)*, Rheum palmatum* (15 g)*, Viola philippica* (10 g)*, Paeonia suffruticosa* (10 g)*, Benincasa hispida* (30 g)*, Prunus persica* (10 g)*,* mirabilite (10 g)	Internal use	Clearing heat, removing dampness	Furuncle carbuncle swelling
	Fuyang Jiming decoctions	*Cinnamomum cassia, Zingiber officinale, Citrus reticulata, Areca catechu, Scrophularia ningpoensis, Scutellaria baicalensis, Coptis chinensis, Angelica sinensis, Areca catechu, Rheum palmatum,* mirabilite	Internal use	Clearing heat, purgative	Asthma, white body heat, cold limbs, thirst, constipation
	Fuzi Dahuang decoctions	*Aconitum carmichaelii* (15 g)*, Rheum palmatum* (18 g)*, Leonurus japonicus* (30 g)*, Astragalus membranaceus* (45 g)*,* mirabilite (10 g)	Internal use	Purgative	Spleen and kidney Yang failure, water stop poison more; uremia
	Xiegan decoctions	Concha haliotidis*, Rheum palmatum, Platycodon grandiflorus, Plantago asiatica,* Antelope’s horn*, Saposhnikovia divaricata,* mirabilite	Internal use	Clearing heat	Red eye
	Sanyi Chengqi decoctions	*Rheum palmatum* (12.5 g)*, Houpoea officinalis* (12.5 g)*, Citrus aurantium* (12.5 g)*,* mirabilite (12.5 g), G*lycyrrhiza uralensis* (10 g)	Internal use	Purgative	Typhoid, abdominal distension, dry throat, thirsty, delirium, heartache, red urine, constipation, wet dream, cough, palpitations, madness, eye disease, aphtha, sore throat, bleeding
	Jiawei Taohe Chengqi decoctions	*Prunus persica, Rheum palmatum,* mirabilite, *Glycyrrhiza uralensis, Cinnamomum cassia, Angelica sinensis, Paeonia lactiflora, Biancaea sappan, Carthamus tinctorius*	Internal use	Purgative	Spitting blood
	Dachengqi	*Rheum palmatum* (15 g)*, Houpoea officinalis* (10 g)*, Citrus aurantium* (15 g)*,* mirabilite (10 g)	Internal use	Purgative	Constipation, hot flashes, delirium, thirst
	Jiawei Taohe Chengqi decoctions	*Prunus persica* (3 g)*, Cinnamomum cassia* (10 g)*, Rheum palmatum* (16 g)*,* mirabilite (10 g), G*lycyrrhiza uralensis* (6 g)*, Whitmania pigra whitman* (10 g)	Internal use	Purgative	Heat trespass blood, heat and blood knot
	Wumei Chengqi decoctions	*Rheum palmatum* (9–15 g)*,* mirabilite (6–9 g), H*oupoea officinalis* (6 g)*, Citrus aurantium* (6 g)*, Prunus mume* (6 g)*, Piper nigrum* (2 g)*, Coptis chinensis* (3 g)	Internal use	Purgative	Insect accumulation
	Taoren Chengqi decoctions	*Rheum palmatum* (12 g)*,* mirabilite (6 g), *Prunus persica* (18pieces)*, Angelica sinensis* (6 g)*, Paeonia lactiflora* (6 g)*, Paeonia suffruticosa* (6 g)	Internal use	Clearing heat	Blood stasis caused by plague day and night fever; night heat day cool
	Taohe Chengqi decoctions	*Prunus persica* (50pieces)*, Rheum palmatum* (200 g)*, Cinnamomum cassia* (100 g)*, Glycyrrhiza uralensis* (100 g)*,* mirabilite (100 g)	Internal use	Clearing heat	Irritability delirium, urination self-interest, nighttime fever, blood stasis, amenorrhea, reproductive system diseases, obstructive intestinal diseases, cerebral hemorrhage diseases
	Banxia Shiwei decoctions	*Pinellia ternata* (250 g)*, Zingiber officinale* (150 g)*, Tetradium ruticarpum* (100 g)*, Cinnamomum cassia* (50 g)*, Atractylodes macrocephala* (150 g)*, Asarum heterotropoides* (150 g)*, Bupleurum longiradiatum* (150 g)*, Paeonia suffruticosa* (150 g)*, Rheum palmatum* (250 g)*,*mirabilite (100 g)	Internal use	Clearing heat	Fever, hard heart and abdomen, bone pain, bitter mouth, vomiting, constipation, crazy words, heat in the heart, jaundice
	Zhijing decoctions	Cicada slough*, Scutellaria barbata, Scolopendra subspinipes mutilans, Beauveria bassiana, Buthus martensii, Pheretima aspergillum, Arisaema cum bile, Typhonium giganteum, Glycyrrhiza uralensis, Hansenia weberbaueriana, Pueraria edulis, Paeonia lactiflora, Scutellaria baicalensis, Rheum palmatum,* mirabilite	Internal use	Clearing heat, removing dampness	Blood deficiency spasm, hand and foot convulsions, mouth and eye skew, angular arch reflex
	Jiziqing drinks	Egg (2pieces)*,* mirabilite (10 g)*,* gypsum rubrum (10 g)	Internal use	Clearing heat, purgative	Delirium, constipation caused by fever
Pulvis	Shuangjie pulvis	*Saposhnikovia divaricata, Rheum palmatum, Mentha canadensis, Paeonia lactiflora, Angelica sinensis, Glycyrrhiza uralensis, Atractylodes macrocephala,* talcum*,* gypsum*, Gardenia jasminoides, Platycodon grandiflorus, Forsythia suspensa, Ligusticum sinense, Nepeta cataria, Ephedra sinica,* mirabilite, S*cutellaria baicalensis*	Internal use	Purgative	Eye swelling pain, acute conjunctivitis
	Fangfeng pulvis	*Leonurus japonicus, Saposhnikovia divaricata, Platycodon grandiflorus, Schisandra chinensis, Anemarrhena asphodeloides Bunge, Scrophularia ningpoensis, Rheum palmatum, Asarum heterotropoides,* mirabilite, *Plantago asiatica, Scutellaria baicalensis*	Internal use	Clearing heat	Eye cataract
	Liangge pulvis	Mirabilite (600 g)*, Rheum palmatum* (600 g)*, Gardenia jasminoides* (300 g)*, Forsythia suspensa* (1250 g)*, Scutellaria baicalensis* (300 g)*, Mentha canadensis* (300 g)*, Glycyrrhiza uralensis* (600 g)*, Lophatherum gracile* (7pieces)	Internal use	Clearing heat	Irritability, facial fever, dizziness, dry throat, swollen tongue, sore throat, red eyes, epistaxis, sore mouth and tongue, sticky saliva, restless sleep, delirium, constipation, red urine, infantile convulsions
	Fangfeng Tongsheng pulvis	*Saposhnikovia divaricata* (5 g)*, Ligusticum sinense* (5 g)*, Angelica sinensis* (5 g)*, Paeonia lactiflora* (5 g)*, Rheum palmatum* (5 g)*, Mentha haplocalyx* (5 g)*, Ephedra sinica* (5 g)*, Forsythia suspensa* (5 g)*,* mirabilite (5 g), gypsum (10 g), *Scutellaria baicalensis* (10 g)*, Platycodon grandiflorus* (10 g)*,* talcum (30 g), G*lycyrrhiza uralensis* (10 g)*, Nepeta cataria* (2.5 g)*, Gardenia jasminoides* (2.5 g)*, Atractylodes macrocephala* (2.5 g)	Internal use	Clearing heat	Wind and heat syndrome
	Xiegan pulvis	*Angelica sinensis, Rheum palmatum, Scutellaria baicalensis, Anemarrhena asphodeloides, Platycodon grandiflorus, Leonurus japonicus,* mirabilite, *Plantago asiatica, Saposhnikovia divaricata, Paeonia lactiflora, Gardenia jasminoides, Forsythia suspensa, Mentha canadensis* (equal dose)	Internal use	Clearing heat	Eye cataract
	Jiawei Kaiye pulvis	Realgar (1 g), cinnabar (6 g), E*uchresta japonica* (12 g)*,* borax (6 g), mirabilite (30–60 g), *Belamcanda chinensis* (12 g)	Internal use	Purgative	Phlegm fire knot
Pills	Zhitan Fuling pills	*Poria cocos* (30 g)*, Citrus aurantium* (15 g)*, Pinellia ternata* (60 g)*,* mirabilite (7.5 g)	Internal use	Expectorant, removing dampness	Phlegm dampness caused by arm pain, limb edema
	Yuxian pills	Lead sulfide (100 g), haematitum (100 g), mirabilite (100 g), cinnabar (50 g), alunite (50 g), plumbum rubrum (25 g)	Internal use	Unblocking collaterals	Epilepsy
	Xishen pills	Lead sulfide (100 g), haematitum (100 g), mirabilite (100 g), cinnabar (50 g), borax (50 g), plumbum rubrum (25 g)	Internal use	Unblocking collaterals	Head confused, mental trance
	Daididang pills	*Angelica sinensis* (50 g)*, Manis pentadactyla* (50 g)*, Prunus persica* (60pieces)*, Rheum palmatum* (200 g)*,* mirabilite (50 g), *Rehmannia glutinosa* (50 g)*, Cinnamomum cassia* (15–25 g)	Internal use	Dispel blood stasis	The weakness man accumulates blood
	Xianxiong pills	*Rheum palmatum* (100 g)*, Prunus armenz* (37.5 g)*, Lepidium virginicum* (37.5 g)*,* mirabilite (37.5 g)	Internal use	Purgative	Neck stiffness and abdominal distension caused by fever
Dans	Bixia dans	Cupric carbonate basic, terra flavausta, mirabilite (equal dose)	Internal use	Expectorant, unblocking collaterals	Red eyes, blind eyes
	Qufeng Zhibao dans	*Saposhnikovia divaricata* (75 g)*, Paeonia lactiflora* (75 g)*,* gypsum (50 g), *Scutellaria baicalensis* (50 g)*, Platycodon grandiflorus* (50 g)*, Rehmannia glutinosa* (50 g)*, Gastrodia elata* (50 g)*, Panax ginseng* (50 g)*, Hansenia weberbaueriana* (50 g)*, Heracleum hemsleyanum* (50 g)*, Angelica sinensis* (125 g)*, Ligusticum sinense* (125 g)*,* talcum (150 g), G*lycyrrhiza uralensis* (100 g)*, Gardenia jasminoides* (30 g)*, Atractylodes macrocephala* (65 g)*, Forsythia suspensa* (15 g)*, Nepeta cataria* (15 g)*, Mentha canadensis* (15 g)*, Ephedra sinica* (15 g)*,* mirabilite (15 g), *Coptis chinensis* (15 g)*, Rheum palmatum* (15 g)*, Platycladus orientalis* (15 g)*, Buthus martensii* (15 g)*, Asarum heterotropoides* (15 g)	Internal use	Clearing heat	Wind and heat syndrome
Buccal tablets	Bixue	Mirabilite, gypsum, gypsum rubrum, saltpeter, G*lycyrrhiza uralensis* (equal dose)	Internal use	Clearing heat	Sore throat, mouth and tongue sore, irritability, manic confusion caused by febrile disease
Mixtures	Fufang Hongteng mixtures	*Sargentodoxa cuneata* (30 g)*, Berberis sieboldii* (30 g)*, Paeonia suffruticosa* (12 g)*, Lonicera japonica* (3 g)*,* mirabilite (12 g), R*heum palmatum* (15 g)*, Prunus persica* (15 g)*, Coix lacryma-job* (12 g)*i, Glycyrrhiza uralensis* (3 g)	Internal use	Clearing heat, purgative	Heat toxin accumulates in the intestines, meat rot and blood corruption
External lotions	Kushen external lotions	*Sophora flavescens* (60 g)*,* aluminum potassium sulfate dodecahydrate kalinite (50 g)*, mirabilite* (60 g)*, Piper nigrum* (15 g)*, Artemisia argyi* (15 g)*, Nepeta cataria* (15 g)*, Cnidium monnieri* (30 g)	External use	Clearing heat, removing dampness, antipruritic	Rheumatic heat evil flows into the skin
Granules	Haijinsha	Lygodium japonicum (100 g),ambrum (40 g), mirabilite (100 g), borax (20 g)	External use	Resolving stones	Urinary calculus

Note: The dose of some medicine preparations has not been found or disclosed.

According to our statistics and analysis, the internal use of mirabilite is mainly adopted in preparations. The main roles of mirabilite in each preparation are clearing heat and purgative. Other functions include removing dampness, expectorant, unblocking collaterals, dispelling blood stasis, antipruritic, and resolving stones. The indications of preparations containing mirabilite involve diseases of 12 systems (Table 4). The common disease types and frequencies in descending order are as follows: digestive system diseases (32.26%), mental and behavioral disorders diseases (16.13%), symptoms, signs and clinical and laboratory abnormalities that cannot be classified elsewhere (13.98%), circulatory system diseases (6.45%), infectious diseases (6.42%), traditional Chinese medicine (ethnic medicine) characteristic diseases (5.91%), respiratory system diseases (4.30%), eye and appendage diseases (4.30%), genitourinary system diseases (3.76%), some infectious and parasitic diseases (3.26%), musculoskeletal system and connective tissue diseases (2.15%), and skin and subcutaneous tissue diseases (1.08%). Owing to the significant effect of mirabilite, the preparations containing this mineral have great advantages in the treatment of certain diseases, such as gastrointestinal diseases (Gai et al., 2023) and mental and behavioral disorders (Wang, 2016).

**TABLE 4:** Classification statistics of mirabilite preparation indications.

Classification of disease	Name of diseases (the number in brackets is the number of preparations used to treat the disease)	Number of times used	Percentage (%)
Digestive system diseases	Constipation (12), thirst (8), intestinal obstruction (7), abdominal distension (6), hot knot in the heart (5), abdominal pain (4), sore mouth and tongue (3), Yangming Fushi (3), subcardiac pain (3), cholelithiasis (1), cleft lip and tongue coke (1), bitter mouth (1), vomiting (1), gastrointestinal dryness and heat (1), gastrointestinal heat (1), jaundice (1). heat toxin accumulates in the colon (1), yellow or black tongue coating (1)	60	32.26
Mental and behavioral disorders diseases	Delirium (6), fidgety (4), crazy (3), epilepsy (2), shortness of breath fidgety (1), head confused (1), mental trance (1), dizziness (1), restless sleep (1), infantile convulsions (1), convulsions (1), hand and foot twitching (1), faint limbs (1), convulsions and palpitations (1), angular arch reflex (1), neck rigidity (1), mouth and eye deviation (1), febrile convulsions (1), spasms (1)	30	16.13
Symptoms, signs and clinical and laboratory abnormalities seen that cannot be classified elsewhere	Fever (10), hot flashes (3), wind heat (2), blood deficiency (2), God tired less gas (2), facial fever (1), nasal mucus (1), body fluid deficiency (1), facial white body heat (1), limbs cold (1), night heat and day cold (1), spontaneous sweating aversion to cold (1)	26	13.98
circulatory system diseases	Vomiting blood (2), cerebral hemorrhage (1), epistaxis (1), blood stasis not scattered (1), blood stasis (1), heat invading blood (1), heat knot blood (1), blood stasis and amenorrhea (1), blood storage (1), blood patches (1), meat rot blood corruption (1)	12	6.45
Infectious diseases	Acute biliary tract infection (1), infection after tubal ligation (1), acute pelvic inflammatory (1), intestinal carbuncle (1), acute simple appendicitis (1), pancreatitis (1), acute pancreatitis (1), cholecystitis (1), liver abscess (1), exudative pleurisy (1), tongue swollen throat carbuncle (1) acute conjunctivitis (1)	12	6.42
Traditional Chinese medicine (ethnic medicine) characteristic diseases	Damp heat and blood stasis (1), phlegm fire knot (1), phlegm dampness (1), qi and yin deficiency (1), typhoid sun syndrome (1), qi stagnation (1), liver and gallbladder damp heat accumulation (1), spleen and kidney yang deficiency (1), water stop poison more (1), stagnation (1), wet dream (1)	11	5.91
Respiratory system diseases	Throat swelling and pain (3), laryngeal obstruction (2), asthma (1), asthma and cough (1), dry throat (1)	8	4.30
Eye and appendage diseases	Eyes red (4), cataract (2), blindness (1), eyes edema (1)	8	4.30
Genitourinary system diseases	Urine red (2), enuresis (1), urinary calculi (1), uremia (1), gonorrhea (1), reproductive system diseases (1)	7	3.76
Some infectious and parasitic diseases	Typhoid fever (2), paratyphoid fever (1), epidemic cerebrospinal meningitis (1), encephalitis B (1), worm accumulation (1)	6	3.26
Musculoskeletal system and connective tissue diseases	Arm pain (1), swollen limbs (1), bone and flesh pain (1), bruises (1)	4	2.15
Skin and subcutaneous tissue diseases	Carbuncles and swellings (1), rheumatism and heat flowing into the skin (1)	2	1.08

## 4 Processing methods of mirabilite in Chinese

Shen Nong Ben Cao Jing recorded the processing of mirabilite as being “refined into paste” (Shang, 1981). Zhou Hou Bei Ji Fang (AD 266–420) proposed boiling (Ge, 2005). For the first time, Lei Gong Pao Zhi Lun adopted paper filtration after water flight and powder processing of mirabilite, and this method increased the cleanliness of mirabilite (Lei). Since the Han Dynasty, many medical writings have recorded that mirabilite needs to be sautéd (Lei; Zhao et al., 2022). In the Tang Dynasty, while following the method of refining and boiling, Xian Shou Li Shang Xu Duan Mi Fang (AD841–845) recorded filtering with paper after hot water blistering (Lan, 1957). In the Song Dynasty, Zheng Lei Ben Cao increased the utilization of firing (Tang, 1991). In the Ming Dynasty, Ben Cao Cheng Ya Ban Ji (AD 1647) first proposed the processing of mirabilite with radish and made a detailed record: mirabilite was decocted in water and boiled with radish (Lu, 2016). Ben Cao Meng Quan (AD 1565) divided the processing of mirabilite with and without excipients into weathered saltpeter and Yuanming powder (Chen, 1988). Ben Cao Gang Mu mentioned that licorice can be added to the processing of mirabilite to alleviate the cold characteristic and salty taste of mirabilite (Li, 2014). In the Qing Dynasty, the processing method of mirabilite excipients (radish and licorice) was followed.

After the founding of the People’s Republic of China, the processing method of mirabilite was gradually standardized. For example, in the Pharmacopoeia of the People’s Republic of China (AD 1963), the processing method of mirabilite, “take radish washed and sliced, placed in a pot with water and boiled through, add the Pixiao (mirabilite) to cook, until all dissolved, remove, filter or clarify the upper layer of liquid after pouring out, cooled to the mirabilite precipitation, remove the mirabilite, drying that is obtained, each Pixiao (mirabilite) 100 pounds, with radish 10–20 pounds” (China, 1985). The subsequent editions of the Pharmacopoeia of the People’s Republic of China only simply pointed out “processing” for mirabilite but neither give the standardized methods and techniques for the processing of mirabilite nor formulate unified processing standards. The Chinese medicine tablet processing methods specific for provinces (municipalities and autonomous regions) are shown in Table 5. The data on mirabilite processing methods are inconsistent. Whether the processing requires supplementation with auxiliary materials, such as the 2010 edition of Qinghai Province Code of Concoction of Traditional Chinese Medicines, the 2012 edition of Tianjin Code of Concoction of Traditional Chinese Medicines, the 1986 edition of Yunnan Province Code of Concoction of Traditional Chinese Medicines, and the 2006 edition of Chongqing Code of Concoction of Traditional Chinese Medicines, all of which do not include the addition of radish, is not stipulated. Second, the choice of auxiliary radish varieties is not clear. For example, most of the concoctions are recorded as radish, and a few select red radishes, white radish, and carrot as auxiliary materials. Third, the raw material is confusing, whether it is “natural mirabilite”, “Poxiao”, “crude mirabilite”, or “Pixiao.” Fourth, the effect of processing parameters such as the proportion of raw materials and auxiliary materials, the amount of water, cooking radish time, and adding raw materials after the total cooking time are not uniformly specified. The above factors will affect the quality and clinical efficacy of mirabilite, resulting in the uncontrollable quality of radish-made mirabilite products. Therefore, the research on the processing method of mirabilite should be strengthened to clarify its scientific connotation.

**TABLE 5:** Processing methods of mirabilite in Chinese Pharmacopoeia and local standards.

References	Raw materials	Radish varieties	Mirabilite: radish/kg	The degree of processing
The Pharmacopoeia of the People’s Republic of China (China, 1985)	Pixiao	—	100∶ (10–20)	Wash and slice the radish, put in a pot with water to boil through, add the Pixiao to cook, until all dissolved
National Code of Concoctions of Traditional Chinese Medicines (China, 1988)	Mirabilite	—	100∶20	Wash and slice the radish, put in a pot with water to boil through, add the mirabilite to cook, until all dissolved
Anhui Province Code of Concoction of Traditional Chinese Medicines (Administration, 2020)	Natural mirabilite	Fresh radish	100∶20	Boil the fresh radish slices thoroughly, then take the radish juice and cook with the natural mirabilite until all dissolved
Beijing Code of Concoction of Traditional Chinese Medicines (Administration, 2010)	Poxiao	Red radish	100∶20	Red radish slices placed in a pot with water and decocted for 30–60 min, remove, discard the dregs, and then add the Poxiao to cook, until completely dissolved
Fujian Province Code of Concoction of Traditional Chinese Medicines (Administration, 2013)	Crude mirabilite	—	100∶ (10–20)	Boil the radish slices thoroughly and then cook them with the crude mirabilite until all is dissolved
Guangxi Province Code of Concoction of Traditional Chinese Medicines (Administration, 2007)	Mirabilite	Fresh radish	100∶20	Boil the fresh radish slices thoroughly and then cook with the mirabilite until all dissolved
Guizhou Province Code of Concoction of Traditional Chinese Medicines (Administration, 2005)	Mirabilite	Fresh white radish	100∶50	Boil fresh grated white radish with water for 2–3 h, remove the slag, take the juice, add mirabilite, stir until all dissolved
Henan Province Code of Concoction of Traditional Chinese Medicines (Administration, 2005)	Pixiao	White radish	100∶0.96	Boil the sliced white radish with the Pixiao until all is dissolved
Hunan Province Code of Concoction of Traditional Chinese Medicines (Administration, 2010)	Pixiao	Fresh radish	100∶20	Fresh radish slices placed in a pot with the right amount of water to boil through, remove the slag, and cook with Pixiao, until all dissolved.
Jilin Province Code of Concoction of Traditional Chinese Medicines (Department, 1987)	Pixiao	Large radish	100∶10	Big radish in a pot with the right amount of water to boil through, with the Pixiao to cook, until all dissolved
Jiangsu Province Code of Concoction of Traditional Chinese Medicines (Administration, 2002)	Natural mirabilite	—	100∶20	Boil the radish slices with water in a pot and then cook them with natural mirabilite until all dissolved.
Jiangxi Province Code of Concoction of Traditional Chinese Medicines (Administration, 2009)	Poxiao	—	100∶ (20–30)	Boil the radish slices in water and then cook them with the Poxiao until dissolved
Shandong Province Code of Concoction of Traditional Chinese Medicines (Administration, 1991)	Mirabilite	—	100∶20	Boil thinly sliced radish with water, then pour in the mirabilite and cook until all dissolved.
Shanghai Code of Concoction of Traditional Chinese Medicines (Administration, 2008)	Pixiao	Fresh white radish	100∶30	Fresh white radish thick slices with water to decoct the juice, remove the slag, add the Pixiao, boil until dissolved
Xinjiang Province Code of Concoction of Traditional Chinese Medicines (Region, 2010)	Natural mirabilite	—	100∶20	Thick slices of radish are boiled thoroughly in water, and then boiled with natural mirabilite until all dissolved

Note: “—” indicates that the type and specification are not clearly defined.

## 5 Pharmacological actions of mirabilite

The pharmacological effects of mirabilite mainly focus on anti-inflammatory detumescence; promotion of cell proliferation, wound healing, gastrointestinal motility, and water discharge; regulation of intestinal flora; anti-muscle paralysis; and analgesia. Recent reports also revealed the cholesterol-lowering function of mirabilite and its pharmacological effects on colon cancer (Table 6).

**TABLE 6:** Pharmacological effects of mirabilite.

Pharmacological effects	Mirabilite single drug or combination therapy	Controls	Animals and weight	Model	Dose (concentration) and administration method	Duration of administration	Minimum active dose (concentration)	Results	References
Anti-inflammatory detumescence effect	Mirabilite	Negative: —	SD rats, 200-250g, (male)	The model of acute pancreatitis after ERCP was prepared by injecting meglumine diatrizoate into the pancreaticobiliary duct	35g, external application	24h	—	In the experimental group, the pancreatic structure was clear, the distribution of interlobular ducts was normal, there was no obvious edema, and no necrosis was found	Chen et al. (2020)
		Positive:Indomethacin suppositories (15 mg / kg)							
	Mirabilite +Qingyi decoction	Negative: —	SD rats, (200±10) g, (male)	Rat model of acute necrotizing pancreatitis was induced by retrograde injection of sodium taurocholate into the biliopancreatic duct	35g, external application,10 ml / kg, orally	48h	—	The pathological damage of pancreas was significantly alleviated after treatment	Zhu et al. (2004)
		Positive:Qingyi decoction (10 ml/kg); Mirabilite (35g)							
	Mirabilite	Negative: distilled water	SD rats, (200±20) g, (half male and half female)	Rat model of acute pancreatitis was induced by intraperitoneal injection of L-arginine	3.14g / kg,ig	7d	—	The levels of AMS, LPS, TNF-α and NO in serum were decreased in each experimental group, and the level of IL-10 in serum of mirabilite group was increased	Chen et al. (2021)
		Positive:Taohe Chengqi decoction(17.27g/kg);*Rheum palmatum*(6.29g/kg);*Prunus persica*							
		(1.57g/kg); *Glycyrrhiza uralensis* (3.14g/kg); *Cinnamomum cassia * (3.14g/kg)							
	Mirabilite +*Rheum palmatum*	Negative: —	SD rats, 270-350g	ANP model was established by multi-point injection of 5 % sodium taurocholate into the pancreas	0.015 mg/g,ig	12h	—	The levels of serum amylase in the experimental group were decreased, the levels of serum IL-2 and IL-6 were increased, and the levels of TNF-ɑ were decreased	Hu et al. (2018)
		Positive: Ulinastatin(0.001 mg/g)							
	Mirabilite	Negative: aluminium sheet	SD rats, 180-220g, male	The model of severe acute pancreatitis was established by retrograde injection of 5 % sodium taurocholate into the pancreaticobiliary duct	30g, external application	72h	—	The amount of ascites, serum TNF-α and ascites AMY in the experimental group were significantly lower than those in the control group	Yang et al. (2017)
	Mirabilite	Negative: aluminium sheet	SD rats, 180-220g, male	The rat model of severe acute pancreatitis was established by retrograde pancreaticobiliary duct injection	30g, external application	72h	—	The pathological scores of the experimental group were significantly decreased, and the levels of serum TNF-α and AMY were decreased	Jin et al. (2016)
	Dahuang Mangxiao pulvis	Negative: —	SD rats, (220±30) g	The rat model of periappendiceal abscess was made by cecal ligation and perforation	5g, external application	9d	—	In the experimental group, the thickening of the small intestinal wall, the infiltration of inflammatory cells, the increase of TNF-α secretion and the level of apoptosis were significantly improved	Lu (2017)
	Mirabilite	Negative:distilled water	BALB/C Mice,18-20g, female	Allergic asthma mouse model was prepared by ovalbumin (OVA) sensitization and challenge method	1.0g/kg,ig	7d	—	The total number of eosinophils and lymphocytes in the bronchoalveolar lavage fluid of the experimental group was significantly reduced, and the serum IgE content was significantly reduced	Liu et al. (2015)
	Mirabilite solution	Negative:normal saline	Japanese rabbits, half male and half female	Xylene-induced inflammation model in mice	40%, 20%, 10%, external application	6d	10%	High, medium and low dose of mirabilite solution can significantly reduce the content of Evans blue in the skin of mice	Liu et al. (2012)
		Positive:Tianqi Dieda Rheumatism Ointment(0.25g/cm^2^)							
	Mirabilite	Negative: —	Guinea pig, (240±10) g,half male and half female	7 % glacial acetic acid induced guinea pig acetic acid colitis model	1.68g/kg,ig	7d	—	Mirabilite group can significantly reduce the number of PGE2 and white blood cells in colitis guinea pigs	Li et al. (2012)
		Positive:Sodium sulfate (1.68g/kg)、Sulfasalazine (0.1mg/kg)							
	Mirabilite solution	Negative: —	Big ear New Zealand rabbit, 2.5-3.0kg	Rabbit phlebitis model induced by indwelling needle	10%, external application	6h	—	At 3d, 5d and 7d, the expression of IL-1, IL-6 and TNF-α in the mirabilite external application group was lower than that in the indwelling needle group	Chen et al. (2020)
	Mirabilite solution	Negative:normal saline	Japanese rabbits, half male and half female	20 % mannitol induced rabbit ear phlebitis model	40%、20%、10%, external application(1.5ml/cm^2^)	6d	10%	Large, medium and small doses of mirabilite solution group can significantly improve the pathological changes of phlebitis	Liu et al. (2012)
		Positive:Xiliaotuo Cream(0.25g/cm^2^)							
	Mirabilite solution	Negative: —	Mice,18-22g, male	Xylene-induced inflammation model in mice	0.4g/mL,ig(0.03ml/g)	1.5h	—	Mirabilite and Poxiao have inhibitory effect on ear swelling in mice, and the effect of mirabilite is better than that of Poxiao	Ying et al. (2003)
		Positive:poxiao (mirabilite) solution (0.4g/mL)							
	Mirabilite solution	Negative: normal saline	Mice, 20-22g	Xylene-induced inflammation model in mice	0.4g/mL, ig(0.03ml/g)	1h	—	The ear swelling degree of mice in the normal saline group was higher than that in the Poxiao group and the mirabilite group	Qiao and Zhu (2021)
		Positive:poxiao (mirabilite) solution (0.4g/ml)							
Promoting cell proliferation and wound healing	Mirabilite solution	Negative: normal saline	Wistar rat, 180-200g,	Non-open soft tissue injury model	40%、20%,wet dressing(1.5m l/cm^2^)	5d	20%	The high and low dose mirabilite solution groups could significantly reduce the symptom score, whole blood viscosity, whole blood low shear reduction viscosity and erythrocyte aggregation index of right hind limb injury in rats, and significantly improve the pathological tissue injury	Wei et al. (2011)
		Positive: Tianqi Dieda Rheumatism Ointment (0.25g/cm^2^)							
	Saturated mirabilite solution	Negative:1%DMSO	SD Mice, 200-230g, male	Round skin full-thickness trauma model	Saturated mirabilite solution, external application	14d	—	In the mirabilite solution group, the wound filling and contraction were faster, the neovascularization tissue was more abundant, the number of cells in the tissue was more, and the tissue density was higher	Liu, F. (2018)
	Mirabilite	Negative:1%DMSO	SD Mice,200±2g, female	Skin punch injury model	1mg/mL, 5mg/mL, 10mg/mL, external application	7d	1mg/ml	The wound area of mirabilite 1mg / ml group decreased by 28.87 %, 5mg / ml group decreased by 68.06 %, 10mg / ml group decreased by 79.01 %	Liu (2015)
		Positive:5mg/mlEGF							
Gastrointestinal motility	Mirabilite solution	Negative: —	Mice,20-22g	Small intestine propulsion motion model	0.3g/mL,ig(0.03ml/g)	25min	—	Poxiao, mirabilite and Xuanming powder have obvious promoting effect on small intestine movement in mice.	Ying et al. (2003)
		Positive:0.3g/mLPoxiao(mirabilite) solution; 0.3g/mLXuan Ming powder(mirabilite) solution							
	Mirabilite solution	Negative:normal saline	Mice,20-22g, half made and half female	Small intestine propulsion motion model	Ig (0.03 ml/g)	30min	—	The propulsion rate of the normal saline group was lower than that of the Poxiao group, the mirabilite group and the Xuanming powder group	Qiao and Zhu (2021)
		Positive:Poxiao (mirabilite)solution;Xuan Ming powder (mirabilite)solution							
	Mirabilite	Negative: —	SD Mice,200-250g	Animal model of topical mirabilite after gastrointestinal surgery	45g, external application	48h	—	External use of mirabilite can significantly promote gastrointestinal propulsion function after abdominal surgery in rats	Liu et al. (2006)
	Mirabilite	Negative:normal saline	KM mice, (20±2) g, half made and half female	Constipation model induced by compound diphenoxylate	1.68g/kg, ig	6d	—	The intestinal propulsive degree of mirabilite group was 35.68 ± 8.31, and the intestinal propulsive rate was 84 %, which was significantly higher than that of model group	Li et al. (2012)
		Positive:Sodium sulfate (1.68 g/kg), Maren Runchang pill (3.2g/kg)							
	Mirabilite	Negative:normal saline	Mice,18-22g, half made and half female	Small intestine propulsion motion model	4g/kg, ig	14d	—	The water content of the small intestine in the mirabilite group was 2.95 ± 0.4g, and the intestinal propulsion rate was 44.30 ± 14.68 %	Xiao et al. (1998)
	Mirabilite +raw Rheum palmatum solution	Negative:normal saline	NIH Mice, (20±2) g,half made and half female	Small intestine propulsion motion model	2.0g/mL, ig	20min	—	Raw and cooked rhubarb had no significant effect on gastric residual rate and small intestinal propulsion rate in mice. Rhubarb combined with mirabilite had significant effect on gastric residual rate and small intestinal propulsion rate	Li et al. (2008)
		Positive:raw *Rheum palmatum* , (2.0g/mL); cooked *Rheum palmatum* (2.0g/mL)							
Regulation of intestinal flora	Mirabilite	Negative: —	ICR Mice(male) 18-20g	—	3.9g/25mL;3.9g/100ml, ig	3w	3.9g/100mL	Mirabilite can increase the abundance of intestinal flora in mice, and the abundance of Ruminococcus in the high-dose mirabilite group was significantly increased	Hu et al. (2022)
	Mirabilite	Negative:distilled water	C57BL/6 Mice(male)	—	158.5mg/kg/day;317mg/kg/day;634mg/kg/day, ig	3W	158.5mg/kg/day	Mirabilite significantly up-regulated Eubacterium nodatum group. The abundance of Erysipelatoclostridium and Ileibacterium significantly down-regulated Clostridium sensu. Abundance of the genus stricto 2	Liu et al. (2006)
Cholesterol-lowering effect	Mirabilite	Negative:distilled water	C57BL/6Mice(male)	Hypercholesterolemia model	158.5mg/kg/day;317mg/kg/day;634mg/kg/day,ig	3w	158.5mg/kg/day	In mirabilite group, cholesterol conversion and bile acid synthesis genes were up-regulated, the expression of low-density lipoprotein receptor in liver was increased, the expression of TRB3 in liver was decreased, the phosphorylation level of AKT was enhanced, the production of FGF15 in ileum was decreased, the expression of FGF15 co-receptor KLB in liver was decreased, JNK signal transduction was inhibited, and the transcription of CYP7A1 was increased	Yang (2021)
Anti-colon cancer	Mirabilite solution	Negative:distilled water	APCmin/+Mice、C57BL/6JMice,(20±2)g	—	0.01mL/g,ig	70d	—	The action sites of mirabilite inhibiting colon cancer include amino acid residues Arg-364 and Asp-533, as well as nucleotides TPC-11, DG-112 and DA-113; potential biomarkers are located in related pathways of bile acid metabolism	Sun et al. (2019)
	Mirabilite solution	Negative:distilled water	APCmin/+Mice、C57BL/6JMice,(20±2)g	—	0.1ml/10g, ig	10w	—	The value of Bax / Bcl-2 in the mirabilite group was lower than that in the control group, the level of serum marker CEA was decreased, and the degree of cell disorder was weakened	Sun et al. (2018)
	Mirabilite solution	Negative:distilled water	APCmin/+Mice(male)、C57BL/6JMice(male),(20±2)g	—	0.1ml/10g, ig	10w	—	The cell structure of intestinal cells in the model group was disordered, and some areas showed typical hyperplasia. The intestinal tissue of mice in the mirabilite group showed regional correction and recovery	Zhang et al. (2018)
Analgesic effect	Mirabilite solution	Negative: normal saline	Mice, female	Hot plate method	4g/kg,ig	270min	—	The percentage of pain threshold increase in mirabilite group was 82.15 % at 30 min, 107.70 % at 90 min, 111.30 % at 150 min, 102.30 % at 210 min and 86.78 % at 270 min, which was significantly higher than that in control group	Xiao et al. (1998)
	Mirabilite	Negative: normal saline	Mice,18-22g	Torsion method	4g/kg, ig	12h	—	The occurrence rate of writhing in mirabilite group was 33.33 %, which was significantly lower than that in control group (91.67 %)	Xiao et al. (1998)
	Mirabilite	Negative: distilled water	KM mice,18-22g, half made and half female	The pain model was induced by intraperitoneal injection of 0.6 % glacial vinegar	1.68g/kg, ig	6d	—	The writhing times of mice in the mirabilite group was 4.36 ± 3.28 in 15 min, which was significantly lower than that in the control group	Li et al. (2012)
		Positive: Sodium sulfate, (1.68g/kg), aspirin (0.15g/kg)							

Note: “w” means “week”, “d” means “day”, “h” means “hour”, and “ig” means “intragastric administration”.

### 5.1 Anti-inflammatory detumescence effect

Tseng et al. (2006) found that mirabilite has good anti-inflammatory detumescence effects possibly because it can accelerate lymphatic circulation, enhance the phagocytic function of reticuloendothelial cells, and reduce local leukocyte infiltration and inflammatory response (Jin et al., 2016). The oral administration of mirabilite (Liu et al., 2012; Hu et al., 2018; Chen et al., 2021) or its external application (Zhu et al., 2004; Lu, 2017; Chen et al., 2020) can effectively inhibit the levels of serum AMS, LPS, IL-6, IL-10, TNF-α and NO in rats with acute pancreatitis and weaken the upregulation of TNF-α and NF-κB genes, thereby reducing the degree of pancreatitis and the level of inflammatory factors and protecting the pancreatic tissues (Ramudo et al., 2005; Malleo et al., 2007; Shen et al., 2017; Zeng et al., 2023). Liu et al. (2015) found that the gavage of mirabilite can significantly reduce the total number of eosinophils, lymphocytes, and serum IgE content in the bronchoalveolar lavage fluid of asthmatic mice to inhibit airway inflammation. Li et al. (2012) reported that mirabilite gavage shows an anti-inflammatory effect by significantly reducing the inflammatory response of PGE2 in the serum of colitis guinea pigs. Gou (2016) stated that mirabilite could stimulate the reticuloendothelial system, particularly the appendix and spleen, to enhance its phagocytosis and improve the anti-inflammatory effect. Gault (1993) suggested that the combination of mirabilite and *R. palmatum* can remove harmful substances such as oxygen free radicals and inflammatory factors in the intestine, relax the sphincter, accelerate intestinal peristalsis, increase intestinal mucosal blood flow, and benefit the early repair of pancreatic organs.

### 5.2 Promoting cell proliferation and wound healing

According to Traditional Chinese medicine, the bitter taste of mirabilite can remove heat, and its salty taste can soften hard substances. This mineral is good at eliminating blood stasis and can dredge all kinds of stases. Cell experiments revealed that mirabilite solution can promote the production of cytokines TGF β1 and VEGF-A, thereby accelerating the formation of endothelial cells, lymphocytes in the wound, and fibroblasts (Wei et al., 2011; Liu, 2015; Liu, 2018). Mirabilite can increase the blood flow under mechanical skin wounds and the exchange intensity of Na^+^ and K^+^ in vascular endothelial cells, promoting wound angiogenesis and significantly inhibiting wound tissue edema (Liu, 2015). Yu (2000) reported that mirabilite has good moisture absorption, strong permeability, low freezing point, and heat absorption, which can shrink capillaries and reduce congestion and swelling. Modern molecular biology studies found that sodium sulfate (the main component of mirabilite) can inhibit the outflow of Ca^2+^ from vascular endothelial cells, activate Ras-ERK1/2 signaling pathway, upregulate the expression of VEGF, TGF β1 and PDGF mRNA, promote vascular tissue regeneration under mechanical skin wounds, and accelerate wound healing (Jiang, 1979). Rudge et al. (2007) found that sodium sulfate molecules can act on a variety of cell surface G protein-coupled receptors, initiate intracellular Ras/ERK/Raf/MAPK and other signaling pathways related to tissue regeneration and inflammation inhibition, and promote the proliferation of vascular smooth muscle cells *in vitro*. Sodium sulfate solution can activate macrophages in the wound site and express a large number of Toll-like receptors (TLRs). Various lymphocytes can recognize the marker factors of various pathogenic microorganisms through TLRs, such as bacterial lipopolysaccharide (LPS), GpG-DNA, peptidoglycan, and PAMP molecules, providing a signal source for acquired immunity (Wang et al., 2020). Reginato et al. (2014) found that a low concentration of sodium sulfate has a significant effect on angiogenesis in the skin wounds of reduced nicotinamide adenine dinucleotide phosphate oxidase (NADPH) subunit Rac2 knockout mice, greatly shortening the wound skin healing rate of mice. Stewart et al. (2013) found that sodium sulfate injection has a significant effect on the proliferation and differentiation of endothelial progenitor cells *in vitro*. Brown et al. (2010) found that mirabilite can significantly upregulate the mRNA and protein expression levels of insulin growth factor 1 and insulin growth factor 2 (IGF-2) in the skin wounds of type II diabetic mice, promoting the binding of IGF-2 to IGF-2/6-phosphate mannose receptor, the expression of TGF-β1, and wound repair. Castronuovo et al. (2011) also found that mirabilite can significantly increase the expression of PDGF protein in diabetic foot wound fluid and promote wound healing. Frank et al. (2006) found that mirabilite can significantly increase the expression of inducible nitric oxide synthase in fibroblasts and vascular endothelial cells in skin ulcers, promoting skin wound repair.

### 5.3 Effect on the digestive system

Mirabilite solution can play a role in the treatment of digestive system diseases, mainly by promoting the secretion of vasoactive intestinal peptide in the intestine (Liu, 2015).

#### 5.3.1 Promoting gastrointestinal motility

The salty and bitter tastes and the cold characteristic of mirabilite are the main reason it can eliminate the food accumulation of gastrointestinal tri-jiao and abdominal fullness and pain. After the oral administration of mirabilite solution, its sulfate ions are not easily absorbed by intestinal mucosal villi. Thus, a hypertonic salt solution layer is formed on the upper layer of intestinal endothelium, and a large amount of water accumulates in the intestine. On the one hand, it can wet the intestine and internal moistening; on the other hand, it can relax the intestine, inhibit abnormal intestinal fermentation, restore normal intestinal peristalsis, and increase intestinal defecation ability (Ji, 2002). Yin et al. (2021) and Ying et al. (2003) found that compared with mirabilite, Poxiao (mirabilite) had more impurities and contained a large amount of Mg^2 +^, so its promoting effect on gastrointestinal tract was stronger. Xiao et al. (1998) found that mirabilite can significantly increase the water content of the small intestine in mice, dilute feces, and promote diarrhea. Xie and Huang (1999) found that mirabilite solution can promote the release of vasoactive intestinal peptide, substance P, and motilin to promote gastrointestinal motility. SP is an extremely important gastrointestinal peptide widely distributed in the enteric nervous system and the entire gastrointestinal tract. It is the main excitatory neurotransmitter in the regulation of gastrointestinal motility and has a double contraction effect on the gastrointestinal longitudinal muscle and the circular muscle (Maake et al., 1999). Liu et al. (2006) found that after abdominal surgery in rats, the external application of mirabilite significantly increased the distribution of SP in the myenteric plexus of the gastric antrum and proximal colon, suggesting that this treatment may enhance the excitability of neurons in the gastrointestinal wall and thereby promote gastrointestinal motility by increasing the release of SP in the gastrointestinal tract. Yang (2021) found that mirabilite can promote the conversion of cholesterol to bile acid and accelerate the synthesis of bile acid, which can lead to diarrhea. *Ruminococcus* bacteria directly promote the secretion of short-chain fatty acids, especially butyric acid that helps regulate intestinal motility, mucus production, and other key functions. Oral mirabilite administration can increase the abundance of *Ruminococcus* in the intestinal flora of normal mice, thereby protecting the gastrointestinal mucosa and moistening the intestine (Tomova et al., 2019).

#### 5.3.2 Regulation of intestinal flora

Orally administered mirabilite can regulate intestinal population. Hu et al. (2022) found that mirabilite improved the abundance and diversity of intestinal bacteria in a dose-dependent manner and increased the beneficial bacteria in the intestine. Low-dose mirabilite only slightly increased the abundance of intestinal bacteria in mice and had minimal effect on the intestinal flora. This finding may be due to the different effects of mineral elements in different concentrations of mirabilite solution on the intestinal flora. Although it can improve the intestinal flora, mirabilite can also enhance the disorder of the intestinal flora. Yang (2021) found that after the gavage of mirabilite, the disordered intestinal flora of hypercholesterolemia mice was significantly improved because bile acids can inhibit the growth of specific bacteria in the intestine. Therefore, mirabilite affects the composition of intestinal microflora by promoting the production of bile acids.

### 5.4 Cholesterol-lowering effect

Orally administered mirabilite has a cholesterol-lowering effect. Yang (2021) found that after the intragastric administration of mirabilite, the cholesterol conversion and bile acid synthesis genes of hypercholesterolemic mice were upregulated, the expression of low-density lipoprotein receptor in liver was increased, the expression of TRB3 in liver was decreased, the phosphorylation level of AKT was enhanced, the production of FGF15 in ileum was decreased, the expression of FGF15 co-receptor KLB in liver was decreased, the JNK signal transduction was inhibited, and the transcription of CYP7A1 was increased. This finding proved that mirabilite can reduce the serum total cholesterol level of hypercholesterolemia mice and promote the conversion of liver cholesterol to bile acid. The mechanism may be through improving insulin resistance, reducing the expression of KLB on the surface of liver cell membrane, inhibiting the phosphorylation of downstream JNK, and promoting the transcription of CYP7A1, leading to the accelerated conversion of cholesterol to bile acid in the liver.

### 5.5 Anti-muscle paralysis effect

Barium ions can change the permeability of a variety of cell membranes so that a large number of potassium ions enter the cells, resulting in hypokalemia and causing significant muscle toxicity in smooth muscle cells, skeletal muscle cells, and myocardial cells. It can produce a stimulating effect, causing muscle paralysis, terminating the reaction, and inducing the clinical manifestations of heart rate disorders, atrial fibrillation, conduction block, ventricular fibrillation, cardiac arrest, decreased muscle tone, decreased muscle strength, tendon reflex disappeared, respiratory muscle paralysis, dyspnea, cyanosis, and asphyxia. 10%–30% sodium sulfate solution has a strong antagonistic effect on barium ions and has a significant mitigation effect on limb paralysis, arrhythmia, and dyspnea (Frank et al., 2006).

### 5.6 Anti-colon cancer effect

Mirabilite has anti-colon cancer effect. Lipid metabolism disorders indicate energy metabolism disorders, accelerate tumor proliferation (Yan et al., 2016), and are closely related to colorectal cancer (Cotte et al., 2018). Zhang et al. (2018) found 11 biomarkers related to colon cancer, namely, retinal acetic acid, linoleic acid, 2-hydroxybutyric acid, 15 (S) -HETE, 6-deoxycatasterone, hypoxanthine, L-acetylcarnitine, PC (16:1 (9Z)/0:0), PC (18:4 (9E, 11E, 13E, 15E)/0:0), PE (18:0/0:00), and PE (21:0/0:0). Three metabolic pathways are related to colon cancer, that is, retinol metabolism, propionic acid metabolism, and glycerophospholipid metabolism. After the administration of mirabilite, the levels of some lipid metabolites such as LysoPC (15:0), LysoPC (16: 0), and LysoPC (16:1 (9Z)) returned to normal. Purine metabolism is involved in gene construction and promotes cell survival and proliferation, which contribute to the development of cancer (Pedley and Benkovic, 2017). After the administration of mirabilite, the hypoxanthine levels decreased significantly, indicating mirabilite can prevent colon cancer by regulating lipid metabolism and purine metabolism. In addition, mirabilite can significantly reduce apoptotic factors and inhibit the proliferation of intestinal tumor cells. (Sun et al., 2018; Sun et al., 2019) also found that mirabilite can inhibit colorectal cancer by regulating the distribution of overall metabolic disorders, and bile acid metabolism is its main targeted pathway. The results of network pharmacology showed that the action sites included amino acid residues Arg-364 and Asp-533 and nucleotides TPC-11, DG-112 and DA-113. Metabolomics results showed that potential biomarkers are located in the related pathways of bile acid metabolism, such as taurine, chenodeoxycholic acid, cholic acid, and deoxycholic acid.

### 5.7 Promoting the discharge of water in the body

Mirabilite promotes the discharge of water and liquid in the body. Zhang et al. (2018) statesd that the external application of mirabilite in the chest can form a high permeability state locally, absorb various effusions in the chest through osmotic pressure, and have a therapeutic effect on malignant pleural effusion. Chen and Xu (2008) and Zhang (2019) also statesd that the SO_4_^2−^ in sodium sulfate, the main component of mirabilite, can locally form a high permeability state and thereby promote the discharge of local water.

### 5.8 Analgesic effect

Some scholars stated that the material basis of the analgesic effect of mirabilite may be related to its trace elements and other components (Ying et al., 2003). Pain is caused by the compression of traumatic hematoma or the stimulation of local peripheral nerves by inflammatory substances. Mirabilite can achieve analgesic effect through nerve reflex (Bo et al., 2006; Ye et al., 2013).

## 6 Toxicological effects of mirabilite

Shen Nong Ben Cao Jing lists Poxiao (mirabilite) as a nontoxic product (Shang, 1981). Mirabilite is refined from Poxiao (mirabilite). Most Chinese traditional medical works recorded that mirabilite is nontoxic, and some stated that mirabilite has small toxicity, such as Yao Xing Lun (Zhen, 1983). Yi Jing Xiao Xue recorded sulfur and *Bolboschoenus yagara* fear of Poxiao (mirabilite), one of the “Nineteen fears” songs in traditional Chinese medicine. This ideology means that sulfur and *Bolboschoenus yagara* are not suitable for combination with mirabilite because they will produce severe toxic and side effects or reduce and destroy the efficacy. However, Qingci Xiao and Xiaoping Mao (Mao et al., 1996; Qiao and Zhu, 2021) found that the combination of sulfur, *Bolboschoenus yagara*, and mirabilite only reduced the anti-inflammatory, analgesic, and purgative effects of mirabilite but had no toxic and side effects. With regard to the use of mirabilite, the Pharmacopoeia of the People’s Republic of China only mentioned that “pregnant women should be cautious about using it, and should not be used with sulfur and *Bolboschoenus yagara*” (China, 1985). Some scholars proposed that mirabilite has a certain irritation to the skin (Tian et al., 2015). Zhou et al. (2015) found that patients who received the external application of mirabilite had local skin flushing or itching. Some scholars also stated that mirabilite is nontoxic, and umbilical application had no damage to local skin (Zhu et al., 2009; Lv, 2017). Qiao and Zhu. (2021) and Ying et al. (2003) found no stimulating effect on the external drip of mirabilite solution in the eyes of rabbits. Zhou (2012) applied 500 g of mirabilite external application combined with gastric tube injection of raw *R. palmatum* bubble solution to treat intraabdominal hypertension in patients with acute pancreatitis. Laboratory tests including blood routine, liver and kidney function, and coagulation function were performed. The results showed no adverse reactions and toxic and side effects during clinical treatment, indicating that mirabilite is nontoxic, side effects, safe to use, and has reliable efficacy. Zhou et al. (2007) reported that the oral administration of mirabilite solution caused chemical irritation to intestinal mucosa but had no significant biological damage to intestinal cells. Shen (2017) intraperitoneally injected mirabilite decoction at a dose of 6.738 g/kg in LD_50_ mice, who died after 1 h of administration. The animals showed renal ischemia. Ying et al. (2003) also mentioned that increasing the dose of mirabilite or continuous use can cause a decline in health, and severe cases can cause dehydration.

## 7 Clinical applications of mirabilite

Mirabilite is widely used in clinical treatment of inflammation, edema, wound healing, digestive system diseases, infusion extravasation, hemorrhoids, skin diseases, breast accumulation, muscle paralysis, intestinal preparation before microscopic examination, and other diseases and symptoms (Table 7).

**TABLE 7:** Clinical application of mirabilite.

Disease	Mirabilite state or combination therapy	Experimental subject	Research design	Grouping and number of people		Treatment, method		Course of treatment	Result	References
				Treatment group	Control group	Treatment group	Control group			
Vein Inflammation	Mirabilite saturated solution	132 patients (60 males, 72 females)	Randomized controlled trial	75 cases (37 males, 38 females)	57 cases (23 males, 34 females)	Mirabilite solution, external wet compress	Routine operation	—	The efficacy of the experimental group was significantly better than the control group	Tong et al. (2007)
	10% Mirabilite solution	50 patients (12 males and 38 females).	Randomized controlled trial	29 cases	21 cases	Mirabilite ice bag (3–5 times a day, 20–30 min each time), external application	Ordinary ice compress (3–5 times a day, 20–30 min each time), external application	4 d	The curative effect of the experimental group was significantly better than that of the observation group	Huang and Zhang (2008)
	Mirabilite solution + *Borneolum syntheticum*	68 patients	Randomized controlled trial	34 cases	34 cases	Mirabilite, *Borneolum syntheticum* solution, external application	50% Magnesium sulfate solution, applying hot soaks	—	The efficacy of the experimental group was significantly better than the control group	(Shi., 2008)
Pancreatitis	Mirabilite + Qingyi decoction	36 patients (22 males and 14 females).	Randomized controlled trial	18 cases	18 cases	Conventional treatment + mirabilite (6–8 h replacement once), external application + Qingyi decoction (three times a day), oral or gastric tube injection	Conventional therapy	7 days	The effective rate of the treatment group was significantly higher than that of the control group, and the hospitalization time was significantly shorter than that of the control group	Wu et al. (2007)
	Mirabilite	234patients	Randomized controlled trial	117 cases	117 cases	Mirabilite (replaced every 4 h), external application	—	24 h	The incidence of PEP in mirabilite group was significantly lower than that in blank group (7.7% vs. 26.5%)	Zeng et al. (2023)
	Mirabilite + Qingyi decoction	65 patients (43 males and 22 females).	Randomized controlled trial	33 cases (21 males, 12 females)	33 cases (21males,12females)	Routine intensive care + mirabilite, external application + Qingyi Decoction (once every 8 h), gastric tube injection	Routine intensive care	5-6 days	The remission time of abdominal pain, abdominal distension and other symptoms, hospitalization time and blood amylase recovery time in the observation group were shorter than those in the control group, with fewer complications and lower rate of conversion to surgery	Liu et al. (2006)
	Mirabilite + Qingyi decoction	47 cases (29 males, 18 females).	Randomized controlled trial	24 cases	23 cases	Routine treatment + mirabilite (three times a day), external application + Qingyi decoction (one dose a day), gastric tube into	Conventional therapy	—	The intestinal recovery time, abdominal pain relief time, white blood cells, complications, mortality and average hospitalization days in the observation group were significantly shorter than those in the control group	Ma (2012)
	Mirabilite + raw *Rheum palmatum* soaking solution	78 cases (41 males, 37 females)	Randomized controlled trial	39 cases (20 males, 19 females)	39 cases (21 males and 18 females).	Mirabilite (once a day), external application + raw *Rheum palmatum* soaking solution (twice a day), gastric tube injection or enema	Routine symptomatic treatment	7–10 days	The disappearance time of abdominal distension and abdominal pain and the recovery time of bowel sounds in the treatment group were shorter than those in the control group	Yu et al. (2019)
	Mirabilite + raw *Rheum palmatum* solution	40 cases (29 males, 11 females)	Randomized controlled trial	20 cases (15 males, 5 females)	20 cases (14 males, 6 females)	Conventional treatment + mirabilite (1–2 h replacement), external application + raw *Rheum palmatum* solution (2 times a day), gastric tube injection	Conventional therapy	7–10 days	The total effective rate of the observation group was higher than that of the control group, and the incidence of complications was lower than that of the control group	Hu et al. (2022)
	Mirabilite + Dachengqi decoction	89 cases (68 males, 21 females)	Randomized controlled trial	45 cases (34 males, 11females)	44 cases (34 males, 10 females)	Conventional treatment + mirabilite (2–3 times a day, 1–2 h each time), external application + Dachengqi decoction (6 times a day), gastric tube injection or enema	Conventional therapy	—	The remission time of abdominal pain and distension, recovery time of gastrointestinal function, hospitalization time, hospitalization expenses and complication rate in the treatment group were lower than those in the control group	Li et al. (2013)
Arthritis	Mirabilite	3 cases (2 males and 1 female).	Random experiment	3 cases (2 males and 1 female).	—	Mirabilite (4 times a day), hot compress	—	7-10 days	The joint symptoms of gout were improved	Li and Duan (2010)
	Mirabilite	80 cases (38 males, 42 females)	Randomized controlled trial	40 cases	40 cases	Mirabilite ice pack	Water ice pack	3 days	The length of postoperative hospital stays and CRP at 72 h after operation in the experimental group were significantly lower than those in the control group	Zhong et al. (2021)
Appendiceal abscess	Mirabilite + *Rheum palmatum* + vinegar	52 patients (30 males and 22 females).	Randomized controlled trial	39 cases (21 males and 18 females).	13 cases (9 males and 4 females).	*Rheum palmatum,* mirabilite grinded with vinegar into a paste, external application	Common antibacterial drugs	10 days	The peripheral white blood cell level in the treatment group decreased faster than that in the control group, and the effective rate was higher than that in the control group	Zhang (2001)
	Mirabilite	118 patients (63 males and 55 females).	Randomized controlled trial	58 cases (33 males and 25 females)	60 cases (30 males, 30 females)	Conventional antibiotic treatment + mirabilite external application (once a day, once 12 h)	Conventional antibiotic therapy	10 days	The total effective rate of appendiceal abscess in the treatment group was higher than that in the control group. The reduction rate of appendiceal abscess mass was higher than that in the control group. The average hospitalization time was shorter than that in the control group. C-reactive protein and white blood cell count were better than those in the swelling control group	Liu, J. (2018)
	Mirabilite	80 patients (33 males and 47 females).	Randomized controlled trial	45 cases	35 cases	Antibiotics + mirabilite external application (2–3 times a day, once 2 h)	Antibiotics treatment	—	The effective rate of the treatment group was 100%, and the hospitalization days and hospitalization expenses were significantly lower than those of the control group	Jin and Gao (2015)
	Mirabilite + *Rheum palmatum* + white vinegar	60 patients (17 males and 43 females).	Randomized controlled trial	30 cases (9 males, 21 females)	30 cases (8males, 22females).	Conventional treatment + *Rheum palmatum*, mirabilite, white vinegar into a paste, external application	Conventional therapy	—	The effective rate of the treatment group was 100%, and the hospitalization time was shorter than that of the control group	Ma et al. (2012)
	Mirabilite	238 patients	Randomized controlled trial	184 cases (101 males and 73 females).	54 cases	Antibiotics + mirabilite external application	Anti-biotic	3-5 days	The course of disease in the treatment group was shorter, and the clinical symptoms and signs were improved or even disappeared quickly	Hu and Zhan (1997)
	Mirabilite + Dahuang Mudan decoction	90 patients (47 males and 43 females).	Randomized controlled trial	45 cases (23 males and 22 females).	45 cases (24 males and 21 females).	Conventional symptomatic treatment + antibiotics + mirabilite, external application + Dahuang Mudan decoction (one dose per day), oral administration	Routine symptomatic treatment + antibiotics	—	The total effective rate of the treatment group was significantly higher than that of the control group. The average hospitalization time of the treatment group was significantly shorter than that of the control group	Yang (2010)
Edema	Mirabilite + *Rheum palmatum*	90 patients	Random experiment	30 cases②30 cases③30 cases	—	① *Rheum palmate*: mirabilite = 1 : 1, hoop②1 : 2, hoop③1: 3, hoop (once a day, once an hour	—	5 d	Anal edema in the treatment group were alleviated	Zhang (2019)
	Mirabilite + traditional chinese medicine	36 female patients	Random experiment	36 female patients	—	Mirabilite (1 h each time), external application + Chinese medicine, oral administration + functional exercise (1 time 30 min, 2 times a day)	—	3 months	26 Cases were markedly effective, 10 cases were effective, the effective rate was 100%	Chen and Xu (2008)
	Mirabilite + *Borneolum syntheticum*	86 patients	Randomized controlled trial	43 cases	43 cases	Mirabilite:*Borneolum syntheticum* (100:1), external application	25% Magnesium sulfate, wet dressing + conventional treatment	14 d	The difference of the treatment group was (3.1 ± 0.62) cm on the 5th day, (4.5 ± 0.41) cm on the 7th day, and (5.0 ± 0.76) cm on the 14th day, which was significantly better than that of the control group	Zhen (2016)
	Mirabilite + *Rheum palmatum* + cisplatin	84 patients	Randomized controlled trial	42 cases	42 cases	Mirabilite, *rheum palmatum*, external application + cisplatin, oral	Cisplatin, oral	28 h	The total effective rate of the treatment group (66.67) was higher than that of the control group (54.76%)	Zhang et al. (2021)
Healing of diabetic foot ulcer	Mirabilite	80 cases	Randomized controlled trial	40 cases	40 cases	Mirabilite (twice a day), external application	Initial therapy	14 d	Treatment group had higher effective rate than control group, with lower average hospitalization days and treatment cost compared to control group	Liu et al. (2011)
Postoperative wound healing	Mirabilite + *Rheum palmatum*	100 female patients	Randomized controlled trial	68 cases	32 cases	Mirabilite, *Rheum palmatum*, external application	Conventional treatment	3 days	Treatment group: 30 cases (44%) cured in 1 course, 26 cases (38%) cured in 2 courses, and 12 cases (18%) cured in 3 courses	Wang (2009)
	Mirabilite + *Rheum palmatum*	100 cases of maternal	Randomized controlled trial	50 cases	50 cases	Mirabilite, *Rheum palmatum*, external application	Conventional treatment	—	The nursing satisfaction of the treatment group was 96.00%, which was significantly higher than that of the control group (78.00%)	Nie et al. (2022)
	Mirabilite + *Rheum palmatum*	80 cases of maternal	Randomized controlled trial	40 cases	40 cases	Mirabilite, *Rheum palmatum* (1–2 times a day), external application	Conventional treatment	—	The total effective rate of the treatment group was 95.0%, which was significantly higher than that of the control group, and the time of exudate stopping was earlier than that of the control group	Xu et al. (2020)
	Mirabilite + *Rheum palmatum*	500 cases of maternal	Randomized controlled trial	250 cases	250 cases	Mirabilite、 *Rheum palmatum* (8 h per day), external application	Conventional treatment	3 d	Treatment group had 222 cases of grade A healing (88.8% rate), significantly surpassing the control group	Xu and Guan (2020)
	Mirabilite + *Rheum palmatum*	90 cases of maternal	Randomized controlled trial	45 cases	45 cases	Mirabilite, *Rheum palmatum*, external application	Routine dressing change	7 days	The grade A healing rate of the observation group was 86.7%, and the grade A healing rate of the control group was 55.6%	He (2019)
	Mirabilite + *Rheum palmatum*	50 cases of maternal	Randomized controlled trial	25 cases	25 cases	Mirabilite、*Rheum palmatum* (quaque die), external application	Routine dressing change	—	Avg healing time and post-op complications in observation group significantly shorter (lower) than control group	Li, J. (2019)
	Mirabilite + *Rheum palmatum*	200 cases of maternal	Randomized controlled trial	100 cases	100 cases	Mirabilite、*Rheum palmatum* (quaque die), external application	Routine dressing change + anti-biotic	7 days	After 1-week, surgical incision healing in the treatment group was much better than in the control group	Zeng (2019)
	20% Mirabilite solution	60 patients (47 males and 13 females).	Randomized controlled trial	30 cases (23 males and 7 females).	30 cases (24 males, 6 females)	20% Mirabilite yarn (quaque die), built-in drainage	10% Hypertonic saline gauze (once a day), built-in drainage	21 d	The degree of wound edema, pain and healing time in the treatment group were better than those in the control group	Zhao (2011)
	Mirabilite	48 cases (23 males, 25 females)	Randomized controlled trial	24 cases (11 males and 13 females)	24 cases (12 males and 12females)	Mirabilite (2 times a day, 1 h), external application	Routine dressing change	7 days	Treatment group had higher wound healing rate, fewer dressing changes, and shorter healing and hospitalization time than control group	Zhao et al. (2021)
	Mirabilite + *Rheum palmatum*	60 female patients	Randomized controlled trial	30 cases	30 cases	Ice compress for 1 day + *Rheum palmatum*、mirabilite (10–15 min each time, 4–6 times a day), external application	Intermittent ice compress for 1 day	7 days	In the treatment group of 60 eyes, 57 eyes were grade A healing and 3 eyes were grade B healing. All better than the control group	Li (2019)
	Mirabilite saturated solution	23 cases (13 males and 10 females).	Random experiment	23 cases (13 males and 10 females).	—	Mirabilite, applying hot soaks	—	1-4 days	Hematoma disappeared in 5 cases at 1 day, 9 cases at 2 days, 7 cases at 3 days, and 2 cases at 4 days	Ye and Lu (2013)
	10% Mirabilite solution	170 patients (89 males and 81 females).	Randomized controlled trial	85 cases (44 males, 41females)	85 cases (45 males and 40 females)	Mirabilite ice bag, external application + normal saline 500mL + β-aescin sodium 25mg, intravenous drip (once a day)	Ice pack with water, external application + normal saline 500mL + β-aescin sodium 25mg, intravenous drip (once a day)	3 days	In the test group, pain reduction was noticeable within 72 h after cold compress, and swelling reduction was noticeable after 24 h	Bo et al. (2006)
	Mirabilite	6 patients (2 males and 4 females).	Random experiment	6 patients (2 males and 4 females).	—	Mirabilite (once a day).external application	—	4-14 d	All cured	Zhang et al. (1990)
	Mirabilite + traditional Chinese medicine	80 patients (49 males and 31 females).	Randomized controlled trial	40 cases	40 cases	Conventional therapy + mirabilite、traditional Chinese medicine, external application	Conventional therapy	7 days	The effective rate of treatment group was 92.5%, which was significantly higher than that of control group (75%)	Guan (2009)
	25% Mirabilite solution	40 females	Randomized controlled trial	20 cases	20 cases	Mirabilite ice bag, external application	Conservative treatment + water ice bag, external application	2 days	The pain degree, swelling degree and comfort degree of the treatment group at 1h, 24h and 48 h were better than those of the control group	Li (2014)
Functional disturbance of gastrointestinal tract	Mirabilite + raw *Rheum palmatum* solution	72 patients (33 males and 39 females).	Randomized controlled trial	37 cases (17 males and 20 females).	35 cases (16 males, 19 females)	Cardiology routine treatment + mirabilite, external application + raw *Rheum palmatum* powder solution (3 times a day), oral + raw *Rheum palmatum* powder solution (1 time a day), enema	Cardiology routine treatment + lactulose (3 times a day), oral + soapy water (1 times a day), enema	5–7 days	The recovery time of bowel sounds in the treatment group was significantly lower than that in the control group	Wu et al. (2004)
	Mirabilite + raw *Rheum palmatum* solution	70 patients (39 males and 31 females).	Randomized controlled trial	35 cases (20 males and 15 females).	35 cases (19 males, 16 females)	Conventional therapy + mirabilite, external application + raw *Rheum palmatum* powder solution (once a day), enema	Conventional therapy	7 days	The treatment group had an 88.6% effective rate, significantly higher than the control group. Defecation and exhaust times were significantly lower than the control group	Xu et al. (2016)
	Mirabilite paste	200 patients (110 males and 90 females).	Randomized controlled trial	100 cases (58 males, 42 females)	100 cases (52males, 48females)	Conventional therapy + mirabilite paste once a day). External application	Conventional therapy	3 days	The treatment group had shorter anal exhaust and defecation time compared to the control group. Also, the number of cases of abdominal distension 3 days after the operation was significantly lower in the treatment group	Qin and Pan (2009)
	Mirabilite + *Rheum palmatum*	42 patients (29 males and 13 females).	Randomized controlled trial	21 cases (14 males and 7 females).	21 cases (15 males and 6 females).	Symptomatic treatment + mirabilite, *Rheum palmatum*, external application	Symptomatic treatment	—	In the experimental group, 16 cases (86%) recovered bowel sounds and anal exhaust within 12 h, and the remaining 5 cases recovered within 24 h	Zhou (2011)
	Mirabilite + chewing gum	114 patients (66 males and 48 females).	Randomized controlled trial	57 cases (34 males, 23 females)	57 cases (32 males, 25females)	Mirabilite, external application + gum (three times a day, one piece at a time, 15–20 min), chewing + conventional treatment	Conventional therapy	—	The treatment group had shorter recovery time for postoperative bowel sounds, first anal active exhaust time, and first defecation time compared to the control group	Wu and Li (2016)
	Mirabilite	70 patients (39 males and 31 females).	Randomized controlled trial	35 cases (19 males, 16 females)	35 cases (20males, 15 females)	Symptomatic treatment + mirabilite (once a day, once 6 h), external application	Symptomatic treatment	3 days	The total effective rate of the treatment group was 88.6%, which was significantly higher than that of the control group	Lai et al. (2016)
	Mirabilite	56 patients (36 males and 20 females).	Randomized controlled trial	28 cases (19 males and 9 females).	28 cases (17 males and 11 females).	Conventional treatment + mirabilite (continuous 24h, daily change), external application	Conventional therapy	7 days	The gastrointestinal function of the treatment group was better than that of the control group	Lin and Xu (2018)
	Mirabilite	60 patients (36 males and 24 females).	Randomized controlled trial	30 cases (20 males, 10 females)	30 cases (16males, 14females)	Routine treatment + mirabilite (1–2 times a day), external application + acupoints (3 times a day, 15 min a time), massage	Conventional therapy	7 days	The improvement of gastrointestinal function score in the treatment group was better than that in the control group	Wang and Jiang (2014)
	Mirabilite	90 patients (42 males and 48 females).	Randomized controlled trial	45 cases (21 males, 24 females)	45 cases (21 males, 24 females)	Perioperative treatment + mirabilite (once a day). External application	Perioperative treatment	5 days	The recovery time of bowel sounds and anal exhaust time in the treatment group were significantly shorter than those in the control group	Lv (2017)
	Mirabilite + lactulose	167 cases	Randomized controlled trial	74 cases	3 cases	Mirabilite + lactulose	Lactulose	3 days	The recovery rate of bowel sounds in the experimental group (62.16%) was better than that in the control group (37.63%)	Pan et al. (2021)
Constipation	Mirabilite + *Rheum palmatum*	100 cases (65 males and 35 females)	Randomized controlled trial	50 cases (32 males, 18 females)	50 cases (33 males, 17 females)	Mirabilite、*Rheum palmatum* (1 times a day), external application + acupoint, massage	Conventional therapy	3 days	The scores of constipation symptoms in both groups decreased after treatment, and the decrease in the treatment group was better than that in the control group	Tao (2022)
	Mirabilite + *Rheum palmatum*	50 cases	Randomized controlled trial	25 cases	25 cases	Mirabilite、*Rheum palmatum* (Once a night), apply the navel.	Polyethylene glycol	2 days	The first defecation time of the treatment group was significantly shorter than that of the control group	Wang (2021)
	Mirabilite + *Rheum palmatum*	70 cases	Randomized controlled trial	35 cases (20 males, 15 females)	35 cases (19 males, 16 females)	Mirabilite、*Rheum palmatum*	Conventional therapy	7 days	The scores of Bristol stool character classification and stool frequency in the treatment group were better than those in the control group	Yu (2014)
	Mirabilite + thunder fire wonder moxibustion	71 cases (39 males, 32 females)	Randomized controlled trial	35 cases (20 males, 15 females)	36 cases (19 males, 17 females)	Mirabilite + Chinese medicine (once a day, once 6 h), umbilical + thunder fire needle (once a day, once 20min)	Conventional therapy	7 days	The effective rate of the treatment group (91.67%) was significantly higher than that of the control group (71.43%), and the defecation time was significantly shorter than that of the control group	Li and Liao (2021)
	Mirabilite solution	60 patients (30 males and 30 females)	Randomized controlled trial	30 patients (15 males and 15 females)	30 patients (15 males and 15 females).	Mirabilite (Once a day), enema	Kaisailu, enema	14 days	The efficacy and comfort of enema in the treatment group were better than those in the control group	Liu, Y. (2018)
	Mirabilite *Rheum palmatum* solution	78 patients (42 males and 36 females)	Randomized controlled trial	39 cases	39 cases	Mirabilite *Rheum palmatum* solution, enema	Mirabilite rhubarb, oral medication	—	The effective rate was 100.0% in the treatment group and 94.9% in the control group	(Song, 2016)
Ileus	Mirabilite	36 patients (29 males and 7 females)	Randomized controlled trial	22 cases	14 cases	Conventional therapy + mirabilite, external application	Conventional therapy	12 h	The experimental group had shorter abdominal pain relief and anal exhaust time compared to the control group. Surgery conversion rate decreased. Postoperative infection incidence and hospitalization days were significantly lower in the experimental group	Xie et al. (2007)
	Mirabilite	57 patients (37 males and 20 females)	Randomized controlled trial	20 cases	①19 cases②18 cases	Conventional therapy +500g mirabilite, external application	Conventional therapy + 50 g mirabilite, 450 gSalt, external application ② conventional treatment +500g salt, external application	72 h	The effective rate of the experimental group was 90%, the effective rate of the control group I was 21.05%, and the effective rate of the control group II was 16.67%	Zhang et al. (2007)
	Mirabilite + Chinese medicine	60 patients (31 males and 29 females)	Randomized controlled trial	31 cases (17 males and 14 females)	29 cases (14 males, 15 females)	Routine treatment + mirabilite, umbilical application + Chinese medicine	Conventional therapy	—	The first defecation time, abdominal pain relief time, first anal exhaust time and hospitalization time in the treatment group were significantly shorter than those in the control group	Jing and Gu (2015)
Abdominal distension	Mirabilite + raw *Rheum palmatum*	60 patients (38 males and 22 females)	Rrandomized controlled trial	30 cases (18 males and 12 females)	30 cases (20 males, 10 females)	Routine treatment + mirabilite (Once a day), external application + raw *Rheum palmatum* (3 times a day), jejunum perfusion	Conventional therapy	14 days	The treatment group had better abdominal distension score and intra-abdominal pressure improvement. Recovery time for exhaust and bowel sounds was shorter in the treatment group compared to the control group	Tian et al. (2015)
	Mirabilite	50 patients (83 males and 67 females)	Randomized controlled trial	50 cases (27 males, 23 females) ②50 cases (28 males, 22 females)	50 cases (28 males, 22 females)	Mirabilite, external application + routine nursing ② Mirabilite, external application + comprehensive nursing intervention	External application	—	Postoperative abdominal distension duration, incision drainage, anal exhaust time, ambulation time, HAMA score, HAMD score and NRS score in the treatment group were better than those in the control group	(Zhang., 2013)
	Mirabilite	120 patients (93 males and 27 females)	Randomized controlled trial	60 cases (48 males and 12 females)	60 cases (45 males and 15 females)	Conventional therapy + mirabilite, external application	Conventional therapy	7 days	The treatment group had better abdominal distension relief and a higher effective rate (90%) compared to the control group (77%)	Hou and Yan (2016)
	Mirabilite + *Rheum palmatum* powder	62 patients (40 males and 22 females)	Randomized controlled trial	30 cases (18 males and 12 females)	32 cases (22 males and 10 females)	Conventional gastrointestinal decompression + *Rheum palmatum* powder + mirabilite (2 times a day), nasal feeding	Conventional gastrointestinal decompression + domperidone (2 times a day), nasal feeding	3 days	The anal exhaust and defecation of the patients in the treatment group were significantly advanced	Cao and Wang (2004)
	Mirabilite + *Rheum palmatum*	32 patients (18 males and 14 females)	Randomized controlled trial	16 cases	16 cases	Conventional therapy + mirabilite、*Rheum palmatum*, external application	Conventional therapy	4 days	In the treatment group, abdominal pressure and inflammation indexes decreased significantly, and recovery time of intestinal function, mechanical ventilation time, and ICU time shortened significantly	Wang (2009)
	Mirabilite + *Rheum palmatum* solution	67 patients (42 males and 25 females)	Randomized controlled trial	34 cases	33 cases	Conventional therapy + mirabilite, external application + *Rheum palmatum* solution (once a day), enema	Conventional therapy	3 days	Compared with the control group, the intra-abdominal pressure of the treatment group decreased significantly at 24h, 48h and 72 h	(Xu., 2010)
	Mirabilite + Liqi Tongbian decoction	62 patients (35 males and 27 females)	Randomized controlled trial	32 cases (19 males and 13 females)	30 cases (16 males and 14 females)	Conventional therapy + mirabilite (2–3 times a day), external application + Liqi Tongbian agreement prescription (1 dose a day), gastric tube injection	Conventional therapy	14 days	The intra-abdominal pressure in the treatment group decreased after treatment, and the total effective rate was significantly higher than that in the control group	Sun et al. (2010)
Infusion extravasation	Mirabilite	240 patients (123 males and 117 females)	Randomized controlled trial	120 cases (65 males and 55 females)	120 cases (58 males and 62 females)	Mirabilite, external application	50% Magnesium sulfate. External application	24 h	The cure rate was 81.82% in the treatment group and 45.46% in the control group	Xu and Wei (2008)
	Mirabilite + *Taraxacum mongolicum* solution	60 patients (37 males and 23 females)	Randomized controlled trial	30 cases	30 cases	Mirabilite、*Taraxacum mongolicum* solution (3–4 times a day, 1 time 30 min), gauze pad dipped in external application.	50% Magnesium sulfate, hot and humid compress	—	In the control group, 8 cases (26.7%) were markedly effective, 10 cases (33.3%) were effective, and 12 cases (40%) were ineffective. In the treatment group, 14 cases (40%) were markedly effective, 12 cases (46.7%) were effective, and 4 cases (13.3%) were ineffective	Sun et al. (2009)
Hemorrhoid	Mirabilite + Chinese drugs solution	1002patients (602 males and 400 females)	Random experiment	1002 patients (602 males and 400 females)	—	Mirabilite + Traditional Chinese medicine (boiling), fumigation, hot compress, sitz bath (15–20 min each time, 2 times a day)	—	—	890 cases were cured, 106 cases were effective, 6 cases were ineffective, and the total effective rate was 99.40%	Su et al. (2015)
	Mirabilite solution	80 patients (60 males and 20 females)	Randomized controlled trial	40 patients (30 males and 10 females)	40 patients (30 males and 10 females)	Mirabilite (2 times a day, 1 time a day 10min–30min), external application	50% Magnesium sulfate solution (twice a day, once a day) 10min–30min), external application	5 days	The treatment group was superior to the control group in clinical efficacy, edema regression time and wound healing	(Wei., 2007)
Acne	Mirabilite + Yeju Qushi Decoction	72 patients (27 males and 45 females)	Random experiment	72 patients	—	Mirabilite, external washing + Yeju Qushi Decoction (One dose a day), oral	Conventional therapy	10 days	39 cases were cured, 20 cases were markedly effective, 10 cases were effective, 3 cases were ineffective, the total effective rate was 95.8%	(Bai., 2012)
Milk accumulates, milk distention, mastitis	Mirabilite	60 cases of maternal	Randomized controlled trial	30 cases	30 cases	Fermented dough applied to the right breast + mirabilite applied to the left breast	Traditional treatment of the right breast + fermented dough applied to the left breast	24 h	The effective rate of the Pixiao external application group was 93%. The milk output of the Pixiao external application group was (50.7 ± 11.4) mL/time, and the milk output of the fermented surface external application group was (46.5 ± 13.5) mL/time, which was significantly higher than that of the traditional treatment group	Zou and Li (2008)
	Mirabilite + Gualou Niubang Decoction	98 females	Random experiment	98 females	—	Mirabilite (2 times a day, each time 40 min), fomentation + Gualou Niubang decoction (One dose daily), oral medication	—	7 days	90 cases were cured, 6 cases were improved, 2 cases were not cured, the total effective rate was 97.9%	Xu and Li (2011)
	Mirabilite	120 cases of maternal	Randomized controlled trial	60 cases	60 cases	Mirabilite, external application	Humalactor	3 days	The effective rate of the treatment group was 96.67%, which was significantly higher than that of the control group 66.67%.	Li et al. (2022)
	Mirabilite + traditional Chinese medicine Decoction	140 cases of maternal	Randomized controlled trial	70 cases	70 cases	Mirabilite (quaque die), external application + traditional Chinese medicine decoction (once a day), oral administration	Traditional Chinese medicine decoction (once a day), oral administration	3-5 days	The total effective rate was 97.3% in the treatment group and 59.8% in the control group. The average days of milk returning were 3 days and 7 days respectively	Dai and Liu (2004)
	Mirabilite + manual milk	22 cases of maternal	Random experiment	22 cases of maternal	—	Mirabilite, external application + manual milk	—	3-5 days	All the 22 patients had successful milk regurgitation return	Ye et al. (2013)
	Mirabilite + Mianhuai pulvis	62 cases of maternal	Random experiment	22 cases of maternal	—	Mirabilite (1times a day), external application + Mianhuai pulvis (2 times a day), oral medication	—	5 days	After 5 days, 43 cases improved significantly, 19 cases improved, and the overall success rate was 100%	Jiang (2013)
	Mirabilite	36 females	Random experiment	36females	—	Mirabilite, external application	—	24 h	There were 30 cases of milk returning within 2 days, accounting for 83.3%	Liu (1993)
	Mirabilite + raw *Rheum palmatum*	267 cases of natural childbirth primipara	Randomized controlled trial	134 cases	133 cases	Kangaroo nursing + raw *Rheum palmatum*, mirabilite, external application	Kangaroo nursing	7 days	The time of postpartum milk opening in the observation group was significantly better than that in the control group. The treatment in the observation group could effectively prevent the incidence of breast congestion swelling, milk deposition and acute mastitis	Shen (1997)
Bowel preparation before microscopic examination	Mirabilite solution + Heshuang	92 cases (46 males, 46 females)	Randomized controlled trial	47 cases (27 males and 20 females)	45 cases (19 males, 26 females)	Hesuan (1 time every 15 min), oral administration + mirabilite solution, oral medication	Heshuang (1 time every 15 min), oral medication	—	The treatment group had a 100% effective intestinal cleaning rate, higher than the control group. Adverse reactions in the treatment group were only 2.12%, significantly lower than the control group (17.78%)	Yang and Zhang (2019)
	Mirabilite solution	44 cases (24 males and 20 females)	Randomized controlled trial	22cases (11 males, 11females)	22 cases (13 males, 9 females)	Mirabilite, oral medication	Hesuan, oral medication	—	The treatment group had less fecal water and foam in the intestines compared to the control group	She et al. (2014)
	Mirabilite solution	462 cases	Randomized controlled trial	109 cases	①106 cases②136 cases③111 cases	Mirabilite, oral medication	Compound polyethylene glycol electrolyte powder group ② Mannitol group ③ Magnesium sulfate group, oral medication	—	The rates of intestinal cleaning, adverse reactions, and comfort during medication were not statistically significant in the four groups	Zhu et al. (2013)

### 7.1 Inflammation

#### 7.1.1 Vein inflammation

Tong et al. (2007) reported that the treatment of vein inflammation should be to clear heat, and remove blood stasis. 68 patients with vein inflammation were treated with external application of mirabilite saturated solution, the results showed that the veins in 61 patients became soft and elastic. Huang and Zhang (2008) iced patients with vein inflammation with a 10% mirabilite solution, the effective rate was 85%, which was higher than the 72% of ordinary ice pack external application. Shi (2008) externally applied mirabilite and *Borneolum syntheticum* solution for the treatment of vein inflammation, and the efficacy was significantly better than the 50% magnesium sulfate solution external application. Lu et al. (2018) used New Zealand rabbits to test the effectiveness of mirabilite in preventing mechanical vein inflammation caused by vein indwelling needles, and the results showed that mirabilite can alleviate the inflammatory response of rabbit ear veins.

#### 7.1.2 Pancreatitis

Liu et al. (2006), Ma et al. (2012), and Wu et al. (2007) proposed that pancreatitis belongs to the “spleen heat” of traditional Chinese medicine. Wu et al. (2017) stated that the treatment of acute pancreatitis should be to clear damp and hot. The treatment of pancreatitis with external application of Pixiao (mirabilite)+ Qingyi decoction for internal administration significantly improved the efficiency. Li et al. (2013) treated 89 patients with pancreatitis with the external application of mirabilite and internal administration of Dachengqi decoction, and the effective rate was 97.8%. Some patients with pancreatitis were treated with mirabilite plus raw *R. palmatum* soaking solution, the times for abdominal distension and abdominal pain to disappear and bowel sound to recover were shorter than those of the conventional treatment, and the complication rate was significantly lower than that of conventional treatment (Yu et al., 2019; Hu, 2022). Zeng et al. (2023) externally applied mirabilite to prevent postoperative pancreatitis (PEP) after endoscopic retrograde cholangiopancreatography. The incidence of PEP in the observation group was significantly reduced.

#### 7.1.3 Arthritis

Li and Duan (2010) stated that using mirabilite externally could prevent the excessive crystallization and deposition of urate in joints, which could result in inflammation. Yi (2016) reported that combining mirabilite with *Borneolum syntheticum* was an effective treatment for gouty arthritis. Three cases of gouty arthritis were treated by applying hot compress with mirabilite. The results showed that the joint symptoms showed significant improvement. Zhong et al. (2021) treated 40 patients with arthritis by applying mirabilite ice treatment. The results showed that the CRP(C-reactive protein) and hospital stay at 72 h after surgery were significantly lower than those in the common ice pack group.

#### 7.1.4 Appendix abscess

Appendix abscess belongs to the thermal stage of intestinal carbuncle in traditional Chinese medicine (Feng, 1998). Zhang (2001) tested the effectiveness of mirabilite in treating intestinal carbuncle. Mirabilite and *R. palmatum* were ground into a paste with vinegar and applied to the abscess of the appendix. The results showed that the effective rate was 100%, and the decrease rate of peripheral white blood cell level was significantly faster than that in the treatment with common antibacterial drugs. Jin and Gao (2015) and Liu (2018) treated appendix abscess with the topical application of mirabilite plus conventional antibiotics. The results showed that the effective rate was significantly higher than that of conventional antibiotics. The proportion of appendix abscess mass reduction, average length of stay, C-reactive protein, and white blood cell count were all better than those in the treatment using conventional antibiotics. Ma et al. (2012) mixed *R. palmatum* powder and mirabilite in a ratio of 2:1 with white vinegar into a paste to treat appendix abscess, and the results showed the effective rate was 100%. Yang (2010) treated 90 patients with appendix abscess by externally applying mirabilite and Dahuang Mudan decoction. the results showed the effective rate was 93.33%.

### 7.2 Edema

In combination with the conventional cisplatin treatment of patients with malignant pleural effusion, Zhang et al. (2021) employed the external application of mirabilite plus *R. palmatum,* the results showed the total effective rate of patients in the treatment group (66.67%) was significantly higher than that in the control group (54.76%). Zhang (2019) treated 90 patients with edema after hemorrhoid surgery with the ratio of *R. palmatum* and mirabilite mixed with 1:3 band and found that the effective rate and recovery rate were 93.09% and 27.58%, respectively. Chen and Xu (2008) treated 36 patients with upper limb edema after breast cancer surgery with the external application of mirabilite and the internal administration of Chinese herbs such as *Astragalus membranaceus, Atractylodes macrocephala, Polyporus umbellatus, Coix lacryma-jobi,* and *Alisma plantago-aquatica,* the effective rate was 100%. Zhen (2016) mixed mirabilite and *Borneolum syntheticum* at a ratio of 100:1 and externally applied the mixture to patients with lower limb lymphedema. In the treatment group, the difference was (3.1 ± 0.62) cm on day 5, (4.5 ± 0.41) cm on day 7, and (5.0 ± 0.76) cm on day 14. The difference was (2.2 ± 0.48) cm on day 5, (4.0 ± 0.37) cm on day 7, and (4.3 ± 0.53) cm on day 14, indicating significant improvement compared with that in the control group.

### 7.3 Wound healing for trauma swelling and pain

Swelling is caused by capillary rupture, bleeding, increased permeability of blood vessel wall after trauma, and extravasation of intravascular fluid into tissue space (Qi, 2001). Pain is caused by the compression of traumatic hematoma or the stimulation of local peripheral nerves by inflammatory response substances (Ma and Ma, 2012).

#### 7.3.1 Healing of diabetic foot ulcers

On the basis of routine treatment in the control group, Liu et al. (2011) applied 200 g of dry mirabilite to the ulcer wounds of patients with diabetic foot for 14 days. The results showed that the ankle brachial index and common peroneal nerve sensory conduction velocity of the treatment group was significantly better than that of the control group, and the average hospitalization days and average treatment costs were significantly lower than those of the control group.

#### 7.3.2 Postoperative wound healing

Wang (2009) applied 500 g of mirabilite and 100 g of *R. palmatum* to the incision of patients with fat liquefaction after operation for 3 days. The cure rate and incision resection rate were significantly higher than those of the control group. Nie et al. (2022) and Zhao et al. (2021) externally applied *R. palmatum* and mirabilite to the incisions of patients with fatty liquefaction in cesarean delivery. The patients’ nursing satisfaction, Chinese medicine symptom rating score, negative emotion score, economic stress score and other prognostic indicators were better than those in the conventional nursing group. Referring to Incision Management for Abdominal Surgery (Li, 2007), Xu et al. (2020), He (2019), Li (2019) and Zeng (2019) applied mirabilite and *R. palmatum* to the incision of patients after cesarean section and achieved ideal efficacy in wound healing. Zhao (2011) treated postoperative wound healing of perianal abscess with the topical application of Chinese medicine mirabilite gauze for 21 days. The results showed that the degree of wound edema, pain degree, and healing time of patients in the treatment group were superior to those in the control group. Li (2019) applied *R. palmatum* and mirabilite powder to the incision of patients undergoing double eyelid plasty and epicanthus correction. The results showed that the external application of mirabilite and *R. palmatum* powder was beneficial to incision healing and shortened postoperative recovery time. Ye and Lu. (2013) treated 23 cases of traumatic hematoma with wet hot compress with saturated solution of mirabilite and achieved satisfactory results. Bo et al. (2006) treated traumatic swelling pain by applying 10% mirabilite solution ice pack plus intravenous infusion of β-aescuin sodium. Compared with water ice pack, mirabilite ice pack had a significant effect on reducing pain degree within 72 h and swelling degree after 24 h. Zhang et al. (1990) found local bleeding, fluid seepage, or foreign body reaction in the skin bag embedded with the pacemaker or in the incision where the catheter electrode was inserted, resulting in local stasis and swelling, even the skin necrosis, secondary infection, with Pixiao (mirabilite) external treatment after all cured. Donghui Guan (Guan, 2009)used mirabilite + *Borneolum syntheticum + raw R. palmatum + raw Phellodendron chinense* powder was externally applied to treat 40 patients with traumatic lower limb swelling, and the clinical effective rate was 92.5%. Li (2014) used 25% mirabilite ice bag to treat 40 patients with perineal hematoma. The results showed that the degree of pain, swelling, and comfort at 1, 24, and 48 h after operation were better than those when using normal ice bag.

### 7.4 Digestive system disease

#### 7.4.1 Functional disturbance of gastrointestinal tract

“Six fu-organs keeping dredging,” restoring gastric motility, and promoting gastrointestinal peristalsis are fundamental methods to solve gastrointestinal dysfunction (Gao, 2007). Wu et al. (2004) and Xu et al. (2016) treated 72 patients with gastrointestinal dysfunction caused by right heart failure with the external application of Pixiao (mirabilite) and oral *R. palmatum* powder solution or enema. The return time of intestinal sound to normal was significantly lower than that of patients treated by routine cardiology. Qin and Pan (2009) applied mirabilite paste to the lower abdomen of patients after abdominal surgery. The results showed that the time of anal exhaust and defecation was significantly shorter than that of the control group (conventional treatment), and the number of cases of abdominal distension 3 days after operation significantly decreased. Zhou (2011) treated 21 patients with umbilical cord around the abdomen with a mixed bag of *R. palmatum* and mirabilite at a ratio of 1:1. Within 12 h, 16 patients recovered bowel sounds and anal exhaust, accounting for 86%. The remaining 5 patients recovered bowel sounds and anal exhaust within 24 h, and the recovery time of gastrointestinal function was significantly shortened. Wu et al. (2017) smeared 200 g of mirabilite powder around the umbilicus of patients undergoing abdominal surgery while taking xylitol sugar-free gum. The results showed that the recovery time of bowel sounds, the first active anal exhaust time, and the first defecation time all improved in the treatment group. Lai et al. (2016) and Lin and Xu (2018) applied mirabilite powder to the navel, and Wang and Jinag (2014) applied mirabilite powder to the navel combined with Zu-san-li and inner pass point massage to treat patients with gastrointestinal dysfunction in acute pancreatitis. The recovery of gastrointestinal function in the treatment group was significantly better than that in the conventional treatment. Lv (2017) treated patients with gastrointestinal dysfunction after colorectal cancer surgery by sticking mirabilite on the navel. The recovery time of bowel sounds, anal exhaust time, defecation time, and hospitalization time in the treatment group were significantly shorter than those in the conventional treatment group. Pan et al. (2021) used mirabilite combined with lactulose to treat gastrointestinal dysfunction in elderly patients after abdominal surgery. The recovery rate of bowel sounds in the experimental group (62.16%) was better than that in the control group (37.63%). 70.27% of the patients in the experimental group finally had anal exhaust or defecation, and only 46.24% of the control group experienced this phenomenon.

#### 7.4.2 Constipation

Xichun Zhang, one of the representatives of the modern school of Chinese and Western medicine, created XiaoFu TongJie decoction for the treatment of constipation. A large dose of radish was decocted and boiled with Pixiao (mirabilite) for the treatment of constipation (Peng et al., 2016). Tao (2022) and Yu (2014) used the external application of mirabilite and *R. palmatum* to treat orthopedic bedridden patients with constipation, and the symptoms of constipation were significantly improved. Wang (2021) applied mirabilite and *R. palmatum* powder to the umbilicus of patients with constipation, who also received orally administered powder of compound polyethylene glycol electrolyte. This study found that the defecation time of patients was significantly shorter than that of patients taking compound polyethylene glycol electrolyte powder alone. Li and Liao (2021) mixed mirabilite, *R. palmatum, Astragalus membranaceus, Borneolum syntheticum, Houpoea officinalis,* and ethanol into a sticky state and applied it to the navel of 35 patients with diabetes mellitus complicated with constipation. Combined with moxibustion, the treatment lasted for 7 days. The effective rate of the treatment group was 91.67%. Liu (2018) conducted enema on 30 patients with acute myocardial infarction complicated with constipation. The results showed that the efficacy and comfort of enema in the treatment group were better than those in the control group (Kaisailu enema). Song (2016) used mirabilite plus *R. palmatum* liquid to treat 39 patients with constipation after stroke, and the curative effect was 100%. Zhang et al. (2014) also stated the therapeutic effect of mirabilite on constipation.

#### 7.4.3 Ileus

Xie et al. (2007) and Zhang et al. (2007) applied Pixiao (mirabilite) externally to the abdominal wall of patients with intestinal obstruction. The time to relief of abdominal pain, time to anal exhaustion, and number of hospitalization days were all significantly reduced. Shan Jing stated that intestinal obstruction belongs to Chinese medicine abdominal pain, the treatment should be based on relieving constipation by purgation. Mirabilite of 500 g was applied to the navel and combined with traditional Chinese medicine (such as *Atractylodes macrocephala, Cynanchum otophyllum, Bupleurum longiradiatum, Houpoea officinalis,* and *R. palmatum*) for the treatment of simple intestinal obstruction. The first defecation time, abdominal pain relief time, first anal exhaust time, white blood cell recovery time, and hospitalization time in the treatment group were significantly shorter than those in the conventional treatment group (Jing and Gu, 2015).

#### 7.4.4 Abdominal distension

Tian et al. (2015) used mirabilite external application combined with *R. palmatum* liquid gavage or enema to treat patients with acute pancreatitis abdominal distension. The results showed that the relief time of abdominal distension in the observation group was significantly shorter than that in the conventional treatment group. Zhang (2013) applied mirabilite to patients with abdominal distension after abdominal surgery, and the duration of abdominal distension and anal exhaust time was significantly improved. Hou and Yan (2016) applied mirabilite to the periumbilical area of patients with liver cirrhosis and abdominal distension. The abdominal distension relief index of the treatment group was better than that of the conventional treatment group, and the effective rate was 90%. The effective rate of abdominal distension relief in patients with damp-heat cirrhosis was 100%. For patients with abdominal hypertension, Cao and Wang (2004) treated them with nasal feeding mirabilite plus raw *R. palmatum* powder, Wang (2009) treated them with mirabilite plus external application of *R. palmatum*, Xu. (2010) treated them with external mirabilite application, and Sun et al. (2010) treated them with external application of mirabilite combined with nasal feeding Liqi Tongbian decoction. Leng et al. (2016) stated that mirabilite can achieve satisfactory results in controlling IAP (intraabdominal pressure) in critically ill patients.

### 7.5 Infusion extravasation

Xu and Wei (2008) externally applied Pixiao (mirabilite) to 120 patients with infusion extravasation, and the control group received gauze soaked with 50% magnesium sulfate. Swelling subsidence and pain relief were recorded every 2 h, and the treatment group was observed for 24 h. The cure rate of the treatment group was 81.82%, which was significantly higher than the 45.46% of the control group. Sun et al. (2009) applied mirabilite and *Taraxacum mongolicum* solution to the puncture site of 30 patients with infusion extravasation, and the control group applied 50% magnesium sulfate wet and hot compress. The effective rate was 40% in 14 cases, 46.7% in 12 cases, and 13.3% in 4 cases, all of which were significantly higher than that in the control group.

### 7.6 Hemorrhoid

The external application of mirabilite can locally soften hemorrhoids, allowing them to easily peel off (Su et al., 2015). Wei. (2007) decocted, fumigated, and hot-applied the traditional Chinese medicine Pixiao (mirabilite) to hemorrhoids. Among the 1002 patients, 890 were cured, 106 experienced drug efficacy, and 6 experienced no drug effect. The total effective rate was 99.40%. Wang and Xu (2013) applied mirabilite to the wound surface of patients with mixed hemorrhoids after external stripping and internal ligation, and the clinical effect was significant.

### 7.7 Skin diseases

As early as in the Tang Dynasty, Sun Simiao’s Bei Ji Qian Jin Yao Fang (AD 652) had records of using mirabilite to treat dermatitis and skin diseases. The external application of mirabilite decoction in the treatment of children with erysipelas was also recorded in Zi Mu Mi Lu.

#### 7.7.1 Herpes

Herpes belongs to the category of snake strand trauma in traditional Chinese medicine, which is mostly caused by heat toxin. The application of mirabilite can lead the heat toxin to dissipate rapidly from the stool (Liu and Lu, 2021). Fu (2011) tested the effectiveness of mirabilite solution in treating genital herpes, the results showed good therapeutic effects. Peng et al. (2018) also stated that the external wet compress of mirabilite and alum in the treatment of herpes has the characteristics of multicomponent, multitarget, definite curative effect, and less adverse reactions.

#### 7.7.2 Acne

Acne belongs to the category of comedone in traditional Chinese medicine. The purpose of applying mirabilite is to clear heat and detoxification, disperse blood stasis, and eliminate acne. Bai. (2012) treated 72 cases of acne with external washing of mirabilite solution combined with the oral administration of Yeju Qushi Decoction, and 39 cases were cured. Among them, 20 cases were markedly effective, 10 cases were effective, and 3 cases were ineffective. The total effective rate was 95.8%. Li et al (2011) has achieved good results in the treatment of damp-heat acne by external washing with Xiaofan pulvis (mirabilite preparation) combined with the oral administration of Cuochuang decoction or Longdan mixtures.

#### 7.7.3 Common wart

Wu et al. (2007) achieved positive outcomes by immersing verruca vulgaris in a saturated mirabilite solution for 1 month.

### 7.8 Breast accumulation, breast distention, mastitis

The accumulation of breast, which cannot be excreted, can lead to breast distension and mastitis (Xu and Li, 2011). A possible reason why the external application of mirabilite is effective for postpartum milk return is that its main component can absorb the surrounding water with the help of osmotic pressure in a hypertonic environment (Shen, 1997). In addition, the salty taste of mirabilite softens the breasts, and its cold character lowers the temperature of the skin, normalizes the body temperature, opens the mammary ducts, and promotes the discharge of milk, which plays a role in the return of milk (Zou and Li, 2008). Li et al. (2022) applied mirabilite and raw *R. palmatum* to prevent breast accumulation in parturient. The results showed that postpartum milk lactation time in the observation group was significantly better than that in the control group, and the treatment in the observation group could effectively prevent the incidence of breast congestive swelling, milk stasis and acute mastitis. Shuhui Yu (Yu et al., 2006) applied fermented dough to the right breast in the observation group and Pixiao (mirabilite) to the left breast, while the control group treated the right breast with traditional methods and applied fermented dough to the left breast. Results, the effective rate of the group was 93%, which was significantly higher than that of the group and the traditional treatment group. Dai and Liu (2004) used the external application of mirabilite for the treatment of patients with postpartum breast distension and recorded a clinical effectiveness rate of 96.67%. Ye et al. (2013) used the traditional technique of lactation and applied 1000 g of mirabilite external compresses on the breasts for 3–5 days; 22 patients were successfully treated. Jiang (2013) externally applied mirabilite to the breasts and combined it with internal administration of Mianhuai pulvis for 3–5 days. The total effective rate was 100%. Liu (1993) treated postpartum patients with lactation deficiency by externally applying mirabilite, and the clinical effective rate was 88.9%. In the study of Xu and Li (2011), the hot compress of mirabilite and Gualou Niubang decoction showed a total effective rate of 97.9% for the treatment of mastitis. Liao reported that the topical treatment of mastitis with mirabilite plus Jinhuang pulvis could quickly eliminate silting and control infection.

### 7.9 Intestinal preparation before microscopic examination

Mirabilite is a purgation drug, and its purgative effect has been exploited by some scholars for intestinal preparation before microscopic examination (Wu et al., 2009). Yang and Zhang (2019) applied polyethylene glycol electrolyte pulvis combined with 20% mirabilite solutionm, and the results showed that the effective rate of intestinal cleaning before colonoscopy was 100% and the incidence of adverse reactions was 2.12%, which was significantly lower than 17.78% in the control group. She et al. (2014) randomly divided 44 subjects into experimental group and control group. The experimental group was orally given mirabilite solution, and the control group was orally given polyethylene glycol electrolyte pulvis. Before colonoscopy, the amount of residual fecal water in the treatment group was significantly less than that in the control group, and the intestinal foam was significantly less than that in the control group. Zhu et al. (2013) divided 462 subjects into four groups (mirabilite group, polyethylene glycol electrolyte pulvis group, mannitol group, magnesium sulfate group). Before colonoscopy, no significant difference in the excellent and good rate of intestinal cleaning, the incidence of adverse reactions, and the excellent and good rate of comfort during medication was observed in the four groups.

### 7.10 Muscle paralysis

Qing Liu (in Frank et al., 2006) found that sodium sulfate injection has a significant recovery effect on Na^+^–K^+^ pump dysfunction and can treat periodic muscle paralysis. Shuhua Zhu (in Frank et al., 2006) found that mirabilite combined with electric shock had a good effect on myocardial fibrosis. Br Med J (Vecht et al., 1989) found that sodium sulfate solution has a certain remission effect on polio patients. Chengguo Yang (in Frank et al., 2006) confirmed that sodium sulfate solution had a significant remission effect on patients with toxic muscle paralysis, and the effective rate was 93.5%.

## 8 Discussions

Mirabilite is a natural mineral medicine with simple chemical composition (Wang, 2022). Its main component is sodium sulfate decahydrate (Na_2_SO_4_·10H_2_O), and it contains small amounts of sodium chloride, magnesium sulfate, calcium sulfate, and other inorganic salts (Wu, R. et al., 2007). Owing to the lack of knowledge of mineral composition, the ancient Chinese often confused mirabilite with saltpeter. Compared with mirabilite, which refers to the mineral with sodium sulfate as the main component, saltpeter is the mineral with potassium nitrate as the main component (Wei et al., 2022). The pharmacological effect of mirabilite is closely related to its chemical composition. When SO_4_^2−^ in sodium sulfate is applied externally, a hypertonic salt solution is formed locally and prompts the tissues to exudate water *in vitro* (Liu, 2019). It is not easy to be absorbed by the intestinal mucosa when taken orally and forms a hypertonic salt solution in the intestine. While adsorbing a large amount of water, it can expand the intestine, causing mechanical stimulation, promoting intestinal peristalsis, and thus causing a defecation effect (Yu, 2000). Sohn et al. (2009) found that mirabilite solution can also relax the surrounding blood vessels, increase absorption, reduce exudation, promote blood circulation, achieve the effect of removing blood stasis, dispersing knots, detumescence and promoting wound healing through the special reaction of sodium salt and sulfate in mirabilite solution. The physical and chemical properties of mirabilite are also one of the reasons for its pharmacological effects. Mirabilite has good moisture absorption, a low freezing point, and its characteristics of absorbing heat. External application of 10%–20% mirabilite solution can accelerate lymphatic circulation, enhance the phagocytic function of reticular endothelial cells, reduce local leukocyte infiltration, and reduce inflammatory response. At the same time, it can expand local blood vessels, accelerate blood flow, improve microcirculation, enhance the phagocytic ability of monocytes, and promote the absorption and dissipation of inflammation. It has anti-inflammatory and analgesic effects and prevents infection (Wu et al., 2007). Qiao Yin and Zhu (2021) and Ying et al. (2003) added 40% mirabilite solution to the medium containing common pathogenic bacteria, and found that the common pathogenic bacteria were not inhibited, indicating that mirabilite did not have a bacteriostatic effect. Recent studies also found that mirabilite has cholesterol-lowering, anti-colon cancer, regulating intestinal flora, anti-muscle paralysis, and analgesic effects. Despite the many pharmacological studies on mirabilite, some problems remain to be solved and improved. For example, current pharmacological experiments of mirabilite lack studies on its active ingredients, which must be a breakthrough in future experiments. Part of the pharmacological mechanism of mirabilite remains unclear. For example, how mirabilite stimulates the reticuloendothelial system, promotes the proliferation of endothelial cells enhances the phagocytosis of cells, and then improves the mechanism of human immune function is still not fully understood (Fu et al., 2023). The mechanism of mirabilite in regulating intestinal flora, increasing the abundance of intestinal flora, and exerting analgesic effects is still less studied. Relevant technologies such as ICP-MS, LC-MS, and GC-MS can be used to analyze the active components in mirabilite to clarify its pharmacological mechanism. Given that most pharmacological research results are obtained through animal models, their effectiveness cannot be fully proved; therefore, additional clinical trials with confirmatory results are needed (Ning et al., 2016). For example, the cholesterol-lowering and anti-colon cancer effects of mirabilite are still only in the experimental stage and have not been applied in clinical practice.

At present, the processing methods and processing indexes of mirabilite in China have not been unified. Taking the yield of mirabilite as the index, Zhang et al. (1998) used the orthogonal design test method to obtain the best process parameters as follows: per 100 kg of Poxiao (mirabilite), 10 kg of radish, 250 kg of water, decoct for 10 min, filter, and crystallize the filtrate at 2°C–4°C. Zheng et al. (2015) took the mass fraction of magnesium ions in mirabilite as the index and obtained the optimum processing technology by orthogonal test: natural mirabilite powder was dissolved in fivefold volume of water at 40 °C water bath, allowed to stand for 30 min; afterward, 0.1-fold of the amount of radish was added to the supernatant for decoction for 60 min and crystallized at < 4°C for 12 h. In terms of temperature, Zhou (1987) showed that the crystallization rate of mirabilite was the highest at 2°C–4°C. Mao (1984) used the solubility curve of mirabilite to calculate that the optimum crystallization temperature of mirabilite, which was 0°C. The research also focused on how to make the drug clean and moderate the nature of the medicine. Li (1999) proposed that the sweet taste and warm character of radish can neutralize the salty taste and cool character of mirabilite and enhance its effect of purging and softening through the work of radish. According to traditional Chinese medicine, boiling radish, which processes mirabilite, is a warm character, so it can alleviate the cool character of mirabilite. The character and taste here originate from the theory of four characters and five tastes in the traditional Chinese medicine properties. The theory was first recorded in the preface of Shen Nong Ben Cao Jing (Wang and Xue, 2008), stating that medicine has four character: cold, hot, warm, and cool. In general, cold and cool medicine have functions such as clearing heat, detoxifying, cooling blood, and nourishing Yin and are mainly for treating heat diseases. Warm and hot medicine have functions such as warming the spleen and stomach, dispersing cold, and aiding Yang and are mainly for treating cold diseases (Zhao, 2008). The theory of character and taste can reflect the inherent attributes of medicine and guide clinical medication (Li and Zhang, 2023), which is an important attribute of traditional Chinese medicine. When the character of traditional Chinese medicine is too much, which will have an adverse effect on the body. Processing traditional Chinese medicine with opposite properties can effectively solve this problem (Deng, 2017). Jiang et al. (2011) found that after the processing of *Coptis chinensis* by *Tetradium ruticarpum* juice, which has a hot character, the phenolic acids, and alkaloids were increased, alleviating the cold character of *C*. *chinensis*. The study on the changes of chemical components and the mechanism of action before and after the processing of mirabilite is limited. Tibetan medicine work Jing Zhu Ben Cao (Pontso, 2012) stated that the mechanism of adding radish for co-cooking during mirabilite processing is that radish can adsorb the salt of mirabilite, reduce the amount of Na^+^, recrystallize mirabilite, reduce the taste of salty and bitter, and increase the taste of sweet and the character of cool (Tibetan medicine stated that radish is cool character, so it can enhance the cool character of mirabilite). Lu (1993) also reported that processing mirabilite with radish could reduce the content of Na^+^ and the side effects of pure sodium sulfate, which was beneficial to detumescence. Yongxue Zhou (Zhang et al., 2018) proposed that the content of sodium in mirabilite after radish processing decreased slightly, that of calcium and magnesium decreased significantly, and that of potassium increased significantly; no heavy metal lead was detected. Mirabilite absorbs zinc, manganese, and iron in radish, which in turn adsorbs copper, lead, and chromium, in mirabilite. Some scholars questioned the processing of radish with mirabilite. Xu (1986) took the yield of mirabilite as the investigation index and found that the crystallization yield of mirabilite without radish was higher than that of radish itself. The coarse grain of mirabilite is also entrained with toxic impurities, which will affect the effect of treating diseases and cause damage to the human body (He and Wang, 2020). In view of these findings, the processing of mirabilite is extremely important. Advanced analytical methods such as UPLC-Q-TOF-MS should be used to analyze the changes of various inorganic salts and active ingredients before and after the processing of mirabilite. The pharmacodynamic comparative study on the effects of purgation and detumescence before and after the processing of mirabilite deeply reveals the scientific connotation of processing mirabilite with radish, providing the basis for optimizing the processing technology of mirabilite, further standardizing the traditional processing technology of mirabilite, establishing the operation rules of production technology, refines the technical parameters, and formulating the quality standard of radish-made mirabilite.

To date, no pharmacokinetic studies have been performed on mirabilite as a single drug. The absorption, distribution, metabolism, and excretion of mirabilite in the body and the changes in blood concentration with time after taking mirabilite remain unclear. Although pharmacokinetic studies were conducted on mirabilite preparations, such as Dachengqi decoction (Yang, 2012), Dahuang Mudan decoction (Nong et al., 2019), Taohe Chengqi decoction (Fan et al., 2001), these investigations are enough to replace the study of mirabilite as a single drug. Preparations contain many medicinal materials and complex components, and distinguishing which metabolite belongs to mirabilite is difficult. Therefore, the pharmacokinetics study of mirabilite must be strengthened. The chemical composition of mirabilite is not toxic, but its main component sodium sulfate will be metabolized in the body as a small amount hydrogen sulfide that will be hydrolyzed to sulfur ions. Sulfur ions are easily absorbed by digestive tract epithelial cells, reducing the levels of peroxidase horseradish (HRP), catalase (CAT), and dopa oxidase (DO) in the body, causing sulfur poisoning, and affecting the level of oxidative metabolism in the body. Sulfides have a paralytic effect on vascular arteries. The combination of sulfides and heme produces thiohemoglobin, which reduces the oxygen load capacity of the blood and induces severe dyspnea (Liu, F., 2018). Murray et al. (1998) found that 30% sodium sulfate solution can significantly cause Ca^2 +^ outflow and K^+^ influx in neuromuscular cells, which is toxic to neuromuscular cells. In general, the clinical application of mirabilite should not focus on its biological therapeutic effect but also consider the toxic and side effects caused by its metabolism *in vivo*. Therefore, the clinical and experimental research on the toxicology of mirabilite must be strengthened.

The clinical application of mirabilite is highly consistent with the records of traditional Chinese medical works. According to the first record of mirabilite in Shen Nong Ben Cao Jing to the Qing Dynasty, mirabilite was widely used in various internal prescriptions, mainly for the treatment of various heat syndromes and constipation, such as abdominal fullness and pain, stool dryness, intestinal carbuncle swelling, and pain. External application is less used and only sporadically adopted for the treatment of breast carbuncle and skin diseases. In the Shen Nong Ben Cao Jing, Poxiao (mirabilite) can transform 72 kinds of stones. In the Tang Dynasty Shi Yao Er Ya (AD 806), mirabilite was called “Huajinshi;” however, it was less mentioned in clinical reports. Recent research on the external application of mirabilite has gradually increased. Mirabilite has been widely used in various types of inflammation, edema, wound healing, skin, breast, and gastrointestinal diseases. External application is one of the characteristic therapies of traditional Chinese medicine, allowing the drug components to be absorbed through the skin so they can directly reach the disease and perform their role. Relevant literature showed that the external application of mirabilite is common in clinical usage. Mirabilite can externally applied alone or in combination with other treatments. For example, Mao (2023) utilized a single external application of mirabilite for the treatment of foot swelling after closed calcaneal fracture. For combined external applications, Zhang et al. (2022) used mirabilite combined with *Borneolum syntheticum* to treat the early swelling of the middle and lower 1/3 fracture of the closed tibia and fibula. Zhang et al. (2022) employed mirabilite and *Tetradium ruticarpum* to treat pancreatitis and abdominal distension. The external application of mirabilite is also combined with traditional Chinese medicine, such as traditional Chinese medicine prescription (Miao et al., 2023), auricular point pressing (Zhang et al., 2022), acupoint massage (Lin et al., 2023), and warm needle (Gulishaera et al., 2015). With the prevalence of external application of mirabilite, the production of external application bags is gradually reaching perfection (Lu et al., 2012; Wang, 2015). Owing to the barrier effect of the stratum corneum of the skin, the traditional external application method has a low absorption efficiency for the drug. Ultrasonic drug introduction is clinically used to improve the penetration of topical drugs (Yang et al., 2017). At present, the hot and cold methods of mirabilite external application of are not uniform. In China, ice packs combined with mirabilite are known to reduce posttraumatic swelling and pain (Wang et al., 2020; Yu et al., 2009), so the traditional external application is ice compress. For example, Li (2014) proposed that cold compress therapy is one of the commonly used treatments in the early stage of acute trauma. Some scholars also stressed that the external application of mirabilite should be a hot compress, such as Qingyun Ye treatment with mirabilite hot and humid compress for hematoma (Ye and Lu, 2013). Li and Duan (2010) used a mirabilite hot compress to treat gouty arthritis, Xu and Li. (2011) used a mirabilite hot compress to treat acute mastitis, and Wang et al. (2017) used *R. palmatum* combined with mirabilite hot compress to reduce the occurrence of pancreatic leakage in severe acute pancreatitis. They proposed that a hot compress of mirabilite can accelerate the movement of mirabilite molecules according to the thermal principle, promote the absorption of intraabdominal exudate, increase local blood circulation, and improve the therapeutic effect and comfort (Liu et al., 2022). An increasing number of scholars have begun to pay attention to the therapeutic effect of mirabilite on colon cancer. For example, Grosso et al. (2014) found that the occurrence of colon cancer in Mediterraneans is closely related to their diet. Mirabilite can treat colorectal cancer by regulating bile acid metabolism and lipid metabolism. However, no clinical report is available on the treatment of colon cancer with mirabilite. Mirabilite has a strong defecation effect. Some scholars proposed that laxatives promote the excretion principle. Mirabilite should have a good therapeutic effect on various infectious diseases in modern medicine and metabolic diseases such as fatty liver, diabetes, and obesity (Zhou et al., 2007). In clinical use of mirabilite, its internal use should not be decoction because the main component of mirabilite is the sulfate of 10 crystal waters, which is easily soluble in water and will hydrolyze under boiling conditions. Its composition changes, altering its activity (Fan et al., 2001; Nong et al., 2019). Mirabilite is also an inorganic salt. It is fried with other drugs, which can reduce the solubility of saponins, alkaloids, and other active ingredients. In addition, the oral administration of mirabilite easily causes nausea (Wu et al., 2009). At present, only a few studies are available on whether different administration methods such as external application and internal administration of mirabilite affect the clinical efficacy of the disease. Only Yuemei Liu proposed the treatment of constipation with mirabilite. Compared with oral or external administration, the effect of enema with mirabilite solution is more evident. In addition, only a few studies focused on the optimal duration of external application of mirabilite. Only Zhidong Zhang mentioned that the external application of mirabilite for 8 and 12 h had minimal treatment effect. The dose–effect relationship of mirabilite efficacy remains unclear. Liu (2018) found that the external application of high-dose mirabilite solution in the treatment of acute myocardial infarction with constipation sometimes has inferior efficacy over the medium and low doses. The number and frequency of clinical use of mirabilite by many doctors are not the same. At present, no basic consensus has been reached on the optimal dosage and frequency of clinical use of mirabilite. The dose, frequency, and mode of administration of mirabilite affect its dissolution and absorption in the gastrointestinal tract and tissue skin, which largely determine its efficacy in the treatment of diseases (He and Wang, 2020). These subjects must be the focus of further studies on mirabilite.

## 9 Conclusion

The medicinal history of mirabilite in China and its chemical composition, processing methods, pharmacology, toxicology, and clinical application were comprehensively described for the first time. As a natural mineral medicine, mirabilite has a simple chemical composition, mainly containing sodium sulfate decahydrate (Na_2_SO_4_·10H_2_O) with a small amount of sodium chloride, magnesium sulfate, calcium sulfate, and other inorganic salts. Mirabilite is widely used in nine ethnic groups (Han, Dai, Kazak, Manchu, Mongolian, Tujia, Uygur, Yi, Tibetan) and a large number of prescription preparations in China. Since the Ming Dynasty, radish processing has become the mainstream processing method of mirabilite in China. The pharmacological effects of mirabilite include anti-inflammatory detumescence, promoting cell proliferation, wound healing, gastrointestinal motility, and water discharge, regulating intestinal flora; lowering cholesterol, anti-muscle paralysis, anti-colon cancer, and analgesic. External application of mirabilite can cause local skin to become flushed or itching. Its oral administration is toxic to neuromuscular cells, and the sulfur ions of its metabolites will also be toxic to the human body. Mirabilite is widely used in the clinical treatment of inflammation, edema, wound healing, digestive system diseases, infusion extravasation, hemorrhoids, skin diseases, breast accumulation, muscle paralysis, intestinal preparation before microscopic examination, and other diseases and symptoms.

Mirabilite is rich in reserves, easy to obtain, widely used in clinical practice, and has a good application prospect. However, its processing methods, active ingredients, quality control, pharmacokinetics, pharmacology and toxicology mechanisms, and standardized clinical application require further studies. In the future, the following three aspects of research on mirabilite should be strengthened. The first is to standardize the processing of mirabilite and provide a basis for formulating the quality standard of mirabilite. The second is to strengthen the discovery of active components in mirabilite and clarify the mechanism of its pharmacological effects. The third is to carry out standardized dose–effect relationship research to lay the foundation for its clinical application.
