# Correction: The application of mirabilite in traditional Chinese medicine and its chemical constituents, processing methods, pharmacology, toxicology and clinical research

**DOI:** 10.3389/fphar.2026.1946698

**Published:** 2026-08-11

**Authors:** Lianbo Tao, Jiaqing Fu, Fangjie Wang, Yinglian Song, Yi Li, Jingwen Zhang, Zhang Wang

**Affiliations:** 1 College of Ethnomedicine, Chengdu University of Traditional Chinese Medicine, Chengdu, China; 2 State Key Laboratory of Southwestern Chinese Medicine Resources, Chengdu University of Traditional Chinese Medicine, Chengdu, China; 3 College of Pharmacy, Chengdu University of Traditional Chinese Medicine, Chengdu, China

**Keywords:** mirabilite, traditional medicine, processing methods, pharmacology, toxicology, clinical applications

The reference for [Liu et al., 2016] was erroneously written as [Liu, T., Zhang, X., Gao, S., Jing, F., Yang, Y., Du, L., et al. (2016). Exosomal long noncoding RNA CRNDE-h as a novel serum-based biomarker for diagnosis and prognosis of colorectal cancer. Oncotarget 7 (51), 85551–85563. doi:10.18632/oncotarget.13465]. It should be [Zhou YX, Wang Q, Zhang XJ. Clinical Application and Pharmacological Research of Mirabilite. Journal of Shaanxi College of Traditional Chinese Medicine, 2007, (1): 54–55].

The original version of this article has been updated

